# Integrative Pharmacokinetic and Metabolomic Analyses Reveal the Underlying Mechanisms of Metabolic Regulation and Support the Safe Use of Oxolinic Acid in *Micropterus salmoides*

**DOI:** 10.3390/antiox15030283

**Published:** 2026-02-25

**Authors:** Jiayin Yang, Mingxiao Li, Xi Chen, Chao Song, Limin Fan, Liping Qiu, Dandan Li, Huimin Xu, Tiejun Li, Ying Huang, Shunlong Meng

**Affiliations:** 1 College of Fisheries and Life Science, Shanghai Ocean University, Shanghai 200120, China; jiayin010212@163.com; 2 Freshwater Fisheries Research Center, Chinese Academy of Fishery Sciences, Wuxi 214081, China; chenxi@ffrc.cn (X.C.); songc@ffrc.cn (C.S.); fanlm@ffrc.cn (L.F.); qiulp@ffrc.cn (L.Q.); lidandan@ffrc.cn (D.L.); xuhuimin@ffrc.cn (H.X.); 3 Wuxi Fisheries College, Nanjing Agricultural University, Wuxi 214081, China; lxsizj@163.com; 4 Zhejiang Marine Fisheries Research Institute, Zhoushan 316021, China; litiejun1982@126.com; 5 Resources and Environment Research Center, Chinese Academy of Fishery Sciences, Beijing 100141, China

**Keywords:** oxolinic acid, *Micropterus salmoides*, pharmacokinetics, metabolomics

## Abstract

Infections caused by *Aeromonas hydrophila* lead to significant economic losses in *Micropterus salmoides* aquaculture. Prior to the single-dose oral administration via gavage, the antibacterial efficacy of Oxolinic Acid (OXO) against the *Aeromonas hydrophila* strain NJ-35 was initially validated using in vitro assays. Subsequently, this study evaluated the pharmacokinetics, tissue residue depletion, and safety of OXO following a single oral dose (30 mg/kg) via medicated feed. The highest tissue concentration was observed in the kidney (C_max_ 17.99 mg/L), while the muscle, the primary edible tissue, reached 4.54 mg/L. Residues in all tissues declined significantly by 72 h, supporting a withdrawal period of 476 degree-days. Metabolomic and biochemical analyses at peak concentration times revealed OXO-induced oxidative stress. Perturbations in the kidney were primarily associated with amino acid metabolism, whereas the muscle exhibited alterations in both lipid and amino acid pathways. Corresponding changes in oxidative stress markers were also detected. These metabolic disturbances and biochemical shifts gradually resolved as OXO was eliminated. This study confirms the in vitro antibacterial efficacy of OXO, establishes a practical withdrawal period, and demonstrates its overall effectiveness and safety profile in *Micropterus salmoides* under the experimental conditions. The findings provide crucial data for the judicious use of OXO in freshwater aquaculture, highlight transient metabolic effects, and contribute to supporting sustainable farming practices.

## 1. Introduction

Infectious diseases, resulting from toxins and other metabolic byproducts generated by the proliferation of pathogenic or opportunistic pathogenic bacteria within the host, represent one of the leading causes of mortality worldwide. Bacterial diseases are characterized by their rapid transmission and extensive dissemination. Within the aquaculture industry, bacterial infections in fish constitute a significant disease burden and are responsible for substantial economic losses.

*Micropterus salmoides* was introduced to China in the 1980s and has since become an economically important fish species. According to the 2024 China Fishery Statistical Yearbook, the total aquaculture production of *Micropterus salmoides* reached 888,000 metric tons in 2023 [1]. *Aeromonas hydrophila* is a widely distributed opportunistic pathogen in freshwater environments, posing significant threats to both aquaculture (particularly freshwater fish farming) and public health [2,3]. Bacterial enteritis caused by *Aeromonas hydrophila* in *Micropterus salmoides* is characterized by high mortality rates (typically 80–100%) and low spontaneous recovery, necessitating a combined treatment regimen involving external disinfectants and oral antibiotics [4,5].

Oxolinic acid (OXO), a first-generation quinolone antimicrobial agent, exhibits broad-spectrum activity with particularly pronounced efficacy against Gram-negative bacteria. As a veterinary-exclusive antibiotic, it demonstrates superior target specificity compared to broad-spectrum agents like doxycycline and fluoroquinolones that are utilized in both human and veterinary medicine. Specifically developed for aquaculture applications, OXO is clinically effective against major piscine pathogens including *Vibrio anguillarum* [6], *Aeromonas hydrophila* [7], and *Aeromonas salmonicida* [8]. Compared to those critically important antimicrobials used in both human and veterinary medicine, OXO, as an early-generation quinolone agent designated for aquaculture use, is considered to pose a lower risk of directly contributing to antimicrobial resistance against clinically essential antibiotics in humans via the food chain. This assessment is based on its minimal use in human clinical practice and its distinct structural characteristics compared to major antibiotic classes employed in human medicine [9,10].

Although existing pharmacokinetic and residue studies on OXO primarily focus on marine species [11,12,13,14], its kinetic behavior and tissue-specific metabolism in freshwater aquaculture species, particularly commercially farmed fish such as *Micropterus salmoides*, remain underexplored. Nonetheless, its broad-spectrum antibacterial activity—especially against Gram-negative bacteria—suggests potential applicability in treating Gram-negative bacterial infections in freshwater fish. This study aims to investigate the pharmacokinetics of the agricultural antibiotic OXO in the freshwater fish *Micropterus salmoides*, with the goal of expanding its scope of application and establishing appropriate dosing protocols. Furthermore, this study will comprehensively assess the impact of OXO on *Micropterus salmoides* by analyzing changes in physiological indicators and metabolomic profiles. As a first-generation quinolone antibiotic, research into its mechanism of action can provide valuable references for the development and application of subsequent quinolone drugs.

## 2. Materials and Methods

### 2.1. Materials and Reagents

All solvents and chemicals were of HPLC or analytical grade. The oxolinic acid standard (CAS# 14698-29-4, Lot# H1814164, ≥98% purity) was procured from Aladdin (Shanghai, China). Methanol, formic acid, and acetonitrile were supplied by Merck (Darmstadt, Germany), heparin sodium by Shanghai Yuanye Bio-Technology Co., Ltd. (Shanghai, China), and QuEChERS cleanup kits by Chongqing Huapu Biotechnology Co., Ltd. (Chongqing, China). Sample preparation utilized 0.22 μm organic phase filters and 1 mL disposable sterile syringes.

### 2.2. Experimental Materials

All animal experiments were conducted in compliance with national and international ethical guidelines, including the Act on Welfare and Management of Animals. Approximately 500 *Micropterus salmoides* were used in this experiment, purchased from (Suzhou Jinchengfu Biotechnology Co., Ltd., Suzhou, China). The fish had an average body weight of 27.88 ± 5 g and an average body length of 11.35 ± 1 cm. They were acclimated for one week in water maintained at 28 ± 1 °C to adapt to the new environment. During this period, 3–5% of weight feed was provided twice daily at fixed times under a constant photoperiod. Water quality parameters were maintained as follows: pH 7.4 ± 0.2, dissolved oxygen 6–8 mg/L. Two days prior to the formal experiment, the fish were divided into tanks, with each tank containing 200 L of dechlorinated tap water that had been aerated for one week. Each tank housed 40 fish, with a total of 6 tanks per group and 2 groups in total.

### 2.3. Experimental Methods

#### 2.3.1. In Vitro Antibacterial Susceptibility Experimental

Preparation of culture media: Sterilized 3% TSB liquid medium and TSA solid medium were prepared using purified water. The media were autoclaved at 121 °C and 0.1 MPa (15 psi) for 20 min. Immediately after sterilization, the mouth of the TSA bottle was flame-sterilized in a biosafety cabinet next to an alcohol lamp, and the medium was poured into sterile Petri dishes. The dishes were gently swirled to ensure even distribution, covered with lids, and allowed to solidify completely before sealing with parafilm. The plates were then inverted and stored at 4 °C for future use.

Bacterial strain resuscitation: The biosafety cabinet was irradiated with ultraviolet light for 30 min, followed by a 5 min ventilation period after turning off the UV lamp. All surfaces and items to be placed inside (including hands, pipettes, reagent bottles, etc.) were wiped with 75% ethanol. Using a sterile inoculation loop, a small amount of bacterial culture was taken from the glycerol-preserved *Aeromonas hydrophila* strain NJ-35 stored at −80 °C. A “zigzag” streak was performed on the solidified TSA plate, which was then inverted and incubated at 28 °C for 18–24 h. A single, morphologically typical and well-formed colony was selected from the streaks. This colony was transferred with an inoculation loop into a tube containing 5 mL of sterile TSB medium. The tube was placed in a constant-temperature shaker set at 28 °C and 180–220 rpm for incubation. The OD_600_ value was monitored in real time, and incubation was immediately terminated when the OD_600_ reached 0.6–0.7. A standard growth curve was plotted with time as the *x*-axis and OD_600_ value as the *y*-axis to determine the generation time and plateau phase.

Minimum Inhibitory Concentration (MIC) Test: In a sterile 96-well plate, OXO was combined with bacterial suspension in the logarithmic growth phase using TSB medium to achieve final drug concentrations of 64, 32, 16, 8, 4, 2, 1, 0.5, 0.25, 0.125, and 0.0625 μg/mL. The plate was incubated statically at 28 °C for 18–24 h. The well with the lowest drug concentration that completely inhibited visible bacterial growth was recorded as the MIC. The OD value of each well at 600 nm was measured using a microplate reader. A concentration-inhibition curve was plotted with drug concentration (logarithmic scale) as the *x*-axis and OD value as the *y*-axis.

Antimicrobial Susceptibility Testing: Using a sterile spreader, a logarithmic-phase bacterial suspension was dripped onto the center of a TSA plate and evenly spread across the surface. The plate was then left at room temperature to allow the suspension to absorb and dry. A sterile punch was used to create vertical wells in the agar, and the agar plugs were carefully removed. Each well was filled with an equal volume of OXO solutions at concentrations of 10, 5, and 1 μg/mL until the wells were completely filled without overflow. Control wells were filled with an equal volume of sterile PBS. The plates were placed upright in a biosafety cabinet for 1–2 h to allow the solutions to diffuse adequately into the agar. Subsequently, the plates were inverted and incubated at 28 °C for 18–24 h. The diameters of the inhibition zones were measured using a vernier caliper.

#### 2.3.2. Experimental Design of Drug Administration

The trial was divided into a dosing group and a control group. The dosing group was administered a feed homogenate thoroughly mixed with OXO at a concentration at a dosage of 30 mg/kg body weight, using a sterile soft tube and syringe, while the control group received the feed homogenate alone via the same method, with assurance of no regurgitation within 1 min.

#### 2.3.3. Sample Collection

Heparinized whole blood samples (with plasma obtained after centrifugation) along with four replicates each of brain, liver, intestine, kidney, and muscle tissues were collected at 0.5, 1, 1.5, 2, 4, 6, 8, 12, 24, 36, 48, 72, 96, 120, 144, 168, 192, 264, 336, 408, 480, and 552 h post-administration. Based on the pharmacokinetic results, we selected the kidney (the tissue with the highest peak concentration) and muscle (the primary edible part) for biochemical and metabolomic analyses. Sampling was conducted at the control time point, peak concentration time, and elimination phase—specifically, kidney tissues were collected from both experimental and control groups at 8 and 72 h post-administration (n = 8 per group), while muscle tissues were collected at 24 and 72 h post-administration (n = 8 per group) for metabolomic and physio-biochemical analyses. All samples were immediately flash-frozen in liquid nitrogen and subsequently transferred to −80 °C for storage.

#### 2.3.4. Pharmacokinetic Assay

OXO was quantified using the external standard method. The optimized cleanup procedure employed matrix-dispersive solid-phase extraction with 50 mg PSA + 50 mg C18 + 150 mg MgSO4. After natural thawing, samples were minced and homogenized (Shanghai Jingxin Industrial Development Co. Ltd., Shanghai, Chian, Tissuelyser-32L). Exactly 1.00 g of homogenate was weighed into a 15 mL sealed centrifuge tube, followed by addition of 5.0 mL of 2% formic acid-acetonitrile solution and 1.00 g NaCl. The mixture was vigorously vortexed at 2000 rpm for 10 min, then centrifuged at 4000 rpm for 10 min. A 2 mL aliquot of the supernatant was transferred to a QuEChERS cleanup tube (containing 150 mg MgSO_4_, 50 mg C18, and 50 mg PSA) for further purification. After centrifugation at 10,000 rpm for 10 min, the supernatant was filtered through a membrane and subjected to instrumental analysis (Waters xevo TD). Details of the liquid chromatography parameters for the pharmacokinetic experiments are provided in the Supplementary Material (Section S1). The withdrawal period was determined based on the pharmacokinetic curve and the maximum residue limit (MRL = 300 μg/kg) established for OXO in animal-derived foods.

This pretreatment procedure was optimized based on the methodology established by Lei Xiao [15].

#### 2.3.5. Metabolomics Assay

Kidney samples were collected from *Micropterus salmoides* at 8 h and 72 h post-administration and from blank controls (n = 4 each). Muscle tissues were obtained at 24 h and 72 h post-administration, plus blank controls (n = 4 each). The collected samples were thawed on ice, and metabolites were extracted with 80% methanol buffer. Briefly, 50 mg of sample was extracted with 0.5 mL of precooled 80% methanol. The extraction mixture was then stored for 30 min at −20 °C. After centrifugation at 20,000× *g* for 15 min, the supernatants were transferred into a new tube and vacuum dried. The samples were redissolved with 100 μL 80% methanol and stored at −80 °C prior to the LC-MS analysis. In addition, pooled QC samples were also prepared by combining 10 μL of each extraction mixture. All samples acquired by the LC-MS system followed machine orders. Detailed descriptions of the liquid chromatography, mass spectrometry, and metabolomics information analysis parameters are provided in the Supplementary Material (Sections S2, S3, and S4, respectively).

Data analysis was performed based on the fold-change (FC) of metabolite levels between groups (i.e., average treatment group/average control group). Metabolite abundance values were log_2_-transformed to approximate normal distribution, followed by Student’s *t*-test. Multivariate statistical analysis using partial least squares-discriminant analysis (PLS-DA) was applied to obtain the variable importance in projection (VIP) score for each metabolite. Differentially expressed metabolites were identified based on the following criteria: FC ≥ 1.2 or FC ≤ 1/1.2, *p*-value < 0.05, and VIP ≥ 1. For multiple-group comparisons, one-way ANOVA was used to calculate *p*-values, with metabolites satisfying *p* < 0.05 and VIP ≥ 1 considered statistically significant.

The analysis was performed using the MetaboCloud platform provided by LC-Bio (Hangzhou LC-Bio Technology Co., Ltd., Hangzhou, China). The sample groups were designed as follows: K-CON (kidney control), K-T (kidney at peak time, 8 h), K-E (kidney at elimination phase, 72 h), M-CON (muscle control), M-T (muscle at peak time, 24 h), and M-E (muscle at elimination phase, 72 h). The raw metabolomics data generated in this study have been deposited in the National Genomics Data Center, China National Center for Bioinformation, under the BioProject accession number PRJCA051728.

#### 2.3.6. Physiological Indicators Assay

The following physiological indicators were measured: reactive oxygen species (ROS), cytochrome P450 enzyme activity (CYP-ECOD), superoxide dismutase (SOD), catalase (CAT), and malondialdehyde (MDA). After complete thawing at room temperature, tissues were homogenized with zirconia beads in 1× PBS (tissue:PBS = 1:10, *w*/*v*) for 10 min. The homogenate was transferred to centrifuge tubes and centrifuged at 10,000× *g* for 15 min at 4 °C. The resulting supernatant was kept on ice for immediate assay of physiological indicators. All procedures were strictly performed according to the kit manufacturer’s instructions. All assay kits were purchased from Shanghai Enzyme-linked Biotechnology Co., Ltd., Shanghai, Chian.

### 2.4. Data Processing

Pharmacokinetic parameters were calculated from the concentration-time data using DAS 2.0 software. Key parameters included: distribution half-life (t_1_/_2_α), elimination half-life (t_1_/_2_β), area under the curve (AUC), time to peak concentration (Tmax), and maximum concentration (Cmax). The enrichment ratio in each tissue was determined by normalizing the highest enrichment concentration to the administration concentration. Withdrawal period (in degree-days) = Temperature × Withdrawal days. Data were analyzed using IBM SPSS Statistics (Version 26). After confirming the assumptions of normality and homogeneity of variance, a one-way analysis of variance (ANOVA) was performed, followed by Dunnett’s test for post hoc comparisons. The concentration-time curves and physiological indicator activity graphs were generated using Origin (2024), while the differential metabolite analysis, KEGG functional enrichment, interaction networks, and correlation analysis plots were created using the LC-Bio platform [16]. For metabolomics analysis, peak extraction was performed with XCMS (3.7.1), and metabolite identification was conducted using meta X software (1.4.2). The main parameters for XCMS and MetaX are provided in the Supplementary Materials (Tables S3 and S4).

## 3. Results and Discussion

### 3.1. In Vitro Antibacterial Effect

**Figure 1:**
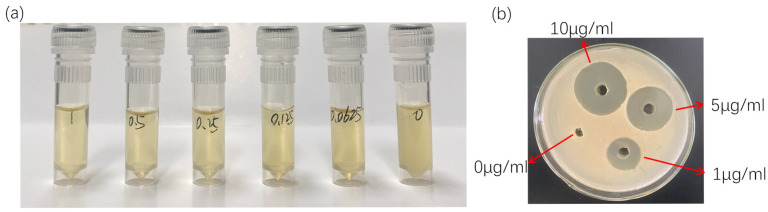
(**a**): Antibacterial effect of different concentrations of OXO against *Aeromonas hydrophila*; (**b**): Drug susceptibility range of *Aeromonas hydrophila* to different concentrations of OXO.

In vitro antibacterial testing of OXO against *Aeromonas hydrophila* NJ-35 demonstrated visible inhibition even at a concentration as low as 0.0625 μg/mL. Based on microplate reader measurements, the bacterial inhibition rate was calculated to be 99.96%, and the susceptibility test showed that the zones of inhibition for OXO at concentrations of 0, 1, 5, and 10 μg/mL were 0, 2.10, 2.68, and 3.21 cm, respectively (Figure 1). Thus, the MIC was determined to be <0.0625 μg/mL.

### 3.2. Pharmacokinetic Analysis

#### 3.2.1. Analysis of Concentration-Time Profile and Pharmacokinetic Parameters

Pharmacokinetics is the discipline that investigates the time course of drug absorption, distribution, metabolism, and excretion (ADME) in living organisms, focusing on how variables such as routes of administration, environmental temperature, and dosage influence kinetic profiles to ultimately optimize dosing regimens [17].

**Figure 2:**
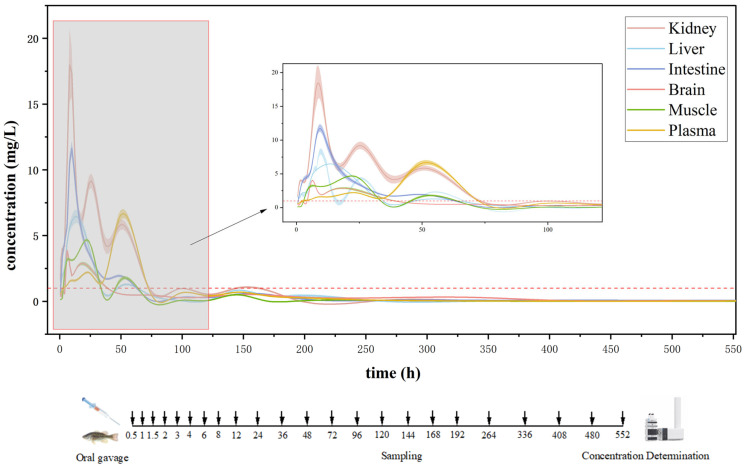
Concentration-time profile of OXO in *Micropterus salmoides* tissues and plasma following oral gavage with 30 mg/kg medicated feed.

Following a single intragastric administration of OXO at 30 mg/kg body weight to *Micropterus salmoides*, we determined its pharmacokinetic parameters in various tissues (Table 1). The LC-MS ion chromatograms are provided in the Supplementary Material (Figure S1). Formulas for calculating pharmacokinetic parameters beyond observed concentration-time curve fitting are provided in the Supplementary Material (Table S1). The method recovery rates of OXO in various tissues of *Micropterus salmoides* are provided in the Supplementary Material (Table S2). Furthermore, we established the corresponding concentration-time profiles (Figure 2), which indicate that the tissue distribution profile of OXO in *Micropterus salmoides* revealed distinct temporal patterns: the brain demonstrated the earliest Tmax at 6 h post-administration with a Cmax of 3.89 mg/L, followed by simultaneous peak concentrations in both kidney and intestine at 8 h (Cmax = 17.99 and 10.64 mg/L, respectively). Subsequent peaks were observed in liver at 12 h (Cmax = 6.42 mg/L), muscle at 24 h (Cmax = 4.54 mg/L), with plasma showing the latest absorption peak at 48 h (Cmax = 6.22 mg/L). The peak concentrations in all tissues exceeded the in vitro minimum inhibitory concentration (MIC) against *Aeromonas hydrophila* strain AH10, 20 strains of *Aeromonas salmonicida*, and 28 strains of *Aeromonas salmonicida* [18,19,20].

Compared with other fish species administered OXO via oral gavage, significant interspecies variations were observed. For example, in lumpfish receiving 25 mg/kg, C_max_ values in head kidney, liver, muscle, and plasma were 4.68, 3.04, 4.01, and 2.12 mg/L, with T_max_ values of 11.1, 9.2, 10.0, and 10.3 h, respectively [13]. Atlantic cod administered 25 mg/kg exhibited a plasma C_max_ of 1.2 mg/L at 24 h [21]. Under conditions of 22 °C and 30 mg/kg dosing in *Sebastes schlegelii*, C_max_ values in serum, muscle, liver, and kidney were 6.80, 9.79, 5.97, and 83.37 mg/L, respectively, all with T_max_ = 6 h except kidney (T_max_ = 1 h). A distinct double-peak phenomenon was noted in the kidney, with an initial peak (83.37 mg/L) at 1 h and a secondary peak (23.64 mg/L) at 6 h [22]. For Atlantic salmon at 28 °C dosed with 25 mg/kg, plasma C_max_ was 0.61 mg/L at 12 h [23]. In turbot maintained at 16 °C and administered 10 mg/kg, serum concentration peaked at 12 h [24]. Observed interspecies variations in OXO pharmacokinetics stem from combined environmental and physiological factors. While water temperature and dosage uniformly influence ADME processes, physiological divergences in metabolic capacity, osmoregulatory strategy, and digestive anatomy are decisive. Specifically, marine teleosts in hyperosmotic environments show reduced renal excretion, potentially prolonging drug half-life. Agastric species like cyprinids, relying solely on intestinal absorption, exhibit distinct uptake kinetics compared to gastric species. Furthermore, compositional and activity differences in hepatic cytochrome P450 isozymes—the primary xenobiotic-metabolizing system—contribute significantly. These factors collectively drive the marked interspecific divergence in OXO pharmacokinetics. A consistent pattern emerged across studies: plasma consistently showed delayed T_max_ and low C_max_, whereas the kidney consistently exhibited early and notably high drug accumulation. These findings suggest that the kidney may play a major role in the metabolism and elimination of OXO in fish.

#### 3.2.2. Pharmacokinetic Profile and Tissue Distribution Overview

In addition to the time to top concentration (T_max_) and the maximum concentration (C_max_), which provide a direct visual representation of drug accumulation in tissues, the area under the concentration-time curve (AUC) and the elimination half-life (t_1_/_2_β) are also critical pharmacokinetic parameters for evaluating efficacy. A relatively high AUC, combined with a suitable elimination half-life, ensures adequate drug residence time for therapeutic effectiveness while simultaneously minimizing the duration of residual presence, thereby reducing potential consumption risks. As summarized in Table 1, the AUC values of OXO in *Micropterus salmoides* across tissues decreased in the order: kidney (477.99 mg/L·h) > intestine (326.81 mg/L·h) > plasma (188.01 mg/L·h) > brain (161.20 mg/L·h) > liver (157.30 mg/L·h) > muscle (85.84 mg/L·h). This distribution profile indicates favorable tissue penetration, with the highest and second-highest levels maintained in the kidney and intestine, respectively—consistent with concentrations required for effective treatment of Gram-negative bacterial infections in the intestinal tract. The comparatively low AUC in muscle tissue suggests minimal residue accumulation, indicating a lower risk in edible parts. Furthermore, the shortest elimination half-life observed in the liver and muscle also supports rapid clearance of OXO from muscle, reinforcing its favorable residue profile. Based on the administered dose of 30 mg/kg in this study, the enrichment ratio of OXO in the tissues of *Micropterus salmoides* was calculated to be highest in the kidney (0.60), followed by the intestine (0.35), liver (0.21), plasma (0.21), muscle (0.15), and brain (0.13).

**Figure 3:**
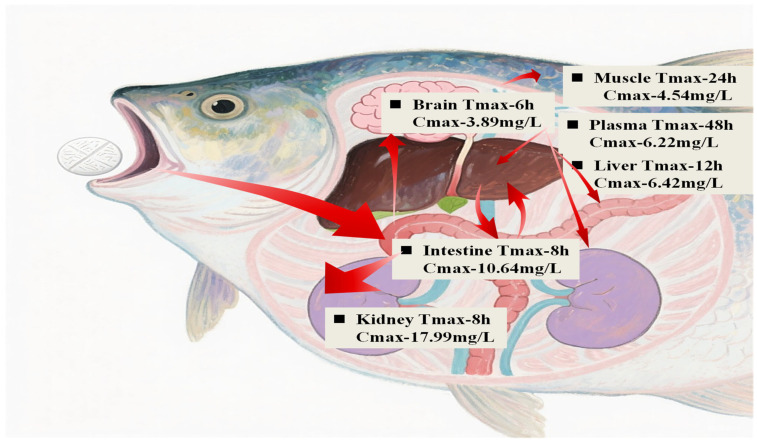
Tissue distribution of OXO in *Micropterus salmoides* after oral gavage of 30 mg/kg.

Due to the oral gavage administration, the intestinal tract was the first site of drug exposure, resulting in an early peak and relatively high concentration. The drug was subsequently distributed to the brain and kidneys, with the latter—as the primary metabolic organ—reaching its peak concentration at 8 h. Subsequent distribution proceeded to the liver and then to muscle tissue (Figure 3). The observed secondary rise in OXO concentrations across multiple tissues at 48 h coincides with the peak plasma concentration, suggesting a redistribution phase from the systemic circulation. Furthermore, enterohepatic circulation likely contributed to the rebound in intestinal drug levels.

The withdrawal period was established in accordance with the maximum residue limit (MRL) of OXO stipulated in the Announcement No. 235 of the Ministry of Agriculture of the People’s Republic of China (Maximum Residue Limits of Veterinary Drugs in Animal Foods) and the NY 5070-2002 standard (Non-Environmental Pollution Food—Maximum Residue Limits for Fishery Drugs in Aquatic Products), which specifies an MRL of ≥300 μg/kg. Based on the pharmacokinetic profiles of OXO in *Micropterus salmoides* obtained in this study, the calculated withdrawal periods for the brain, liver, intestine, kidney, muscle, and plasma were 408 h, 264 h, 192 h, 168 h, 408 h, and 192 h, respectively. All tissues reached residue levels below the MRL by 408 h post-administration. Therefore, it is concluded that the withdrawal period for OXO administered via oral gavage at a dose of 30 mg/kg body weight is 408 h, equivalent to 17 days, 476 degree-days.

### 3.3. Metabolomic and Biochemical Profiling in Kidney and Muscle of Micropterus Salmoides After Oral Gavage of 30 mg/kg

#### 3.3.1. Metabolomic Differential Analysis of Kidney

**Figure 4:**
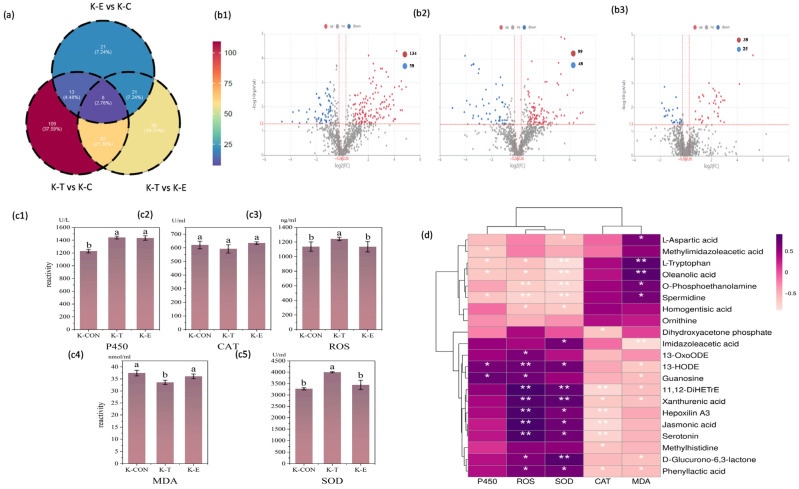
Kidney metabolic profiling and biochemical correlations in *Micropterus salmoides* following oral gavage administration of 30 mg/kg OXO: (**a**) Venn diagram of kidney metabolites; (**b1**) Volcano plot: K-T vs. K-CON; (**b2**) Volcano plot: K-T vs. K-E; (**b3**) Volcano plot: K-E vs. K-CON; (**c1**) P450 activity in kidney groups; (**c2**) CAT activity in kidney groups; (**c3**) ROS activity in kidney groups; (**c4**) MDA content in kidney groups; (**c5**) SOD activity in kidney groups; (**d**) Metabolite-biomarker correlation heatmap in kidney. Data are marked with asterisks to indicate significant differences: **, *p* < 0.01; *, a, b, *p* < 0.05.

This experiment identified key common differential metabolites by comparing the kidney metabolomes among the K-T, K-E, and K-CON following oral administration of OXO via medicated feed. The QC heatmap of kidney metabolomics, intra-group sample reproducibility, and PLS-DA evaluation of inter-group differences in metabolomics are provided in the Supplementary Materials (Figures S2 and S3). The statistics of kidney secondary metabolites across multiple comparison groups and the kidney differential metabolites from individual group comparisons are also available in the Supplementary Materials (Figures S4 and S5). A total of 192 differential metabolites were detected between the K-T and K-CON groups, among which 134 were significantly upregulated and 58 were significantly downregulated (Figure 4(b1)). Between the K-T and K-E groups, 147 differential metabolites were identified, with 99 upregulated and 48 downregulated (Figure 4(b2)). Comparison of the K-E and K-CON groups revealed 63 differential metabolites, including 38 upregulated and 25 downregulated (Figure 4(b3)).Venn analysis was performed to identify common differential metabolites across comparison groups.

Metabolomic analysis revealed distinct patterns of shared metabolites across comparisons(Figure 4a): 21 metabolites were common to both K-T vs. K-CON and K-E vs. K-CON comparisons; 70 metabolites were shared between K-T vs. K-CON and K-T vs. K-E comparisons; 29 metabolites overlapped in K-E vs. K-CON and K-T vs. K-E comparisons; while 8 metabolites were consistently identified across all three comparison sets (K-T vs. K-CON, K-E vs. K-CON, and K-T vs. K-E). These 8 shared metabolites were: Diethyl phthalic acid, OXO, Tinidazole, Dodecyl benzenesulfonate, Quinidine N-oxide, N-(6-Chloro-9h-pyrido [3,4-b]indol-8-yl)-3-pyridinecarboxamide, Oleanolic acid, and 5-(2-Aminopropyl)indole.

#### 3.3.2. Functional Enrichment Analysis of Differential Metabolites of Kidney

Functional enrichment analysis of differential metabolites offers an efficient strategy for predicting biological functions, associated pathways, and their potential relevance to the phenotypic conditions under investigation [25]. Kidney KEGG enrichment analysis and metabolite correlation analysis are provided in the Supplementary Materials (Figures S6 and S7).

**Figure 5:**
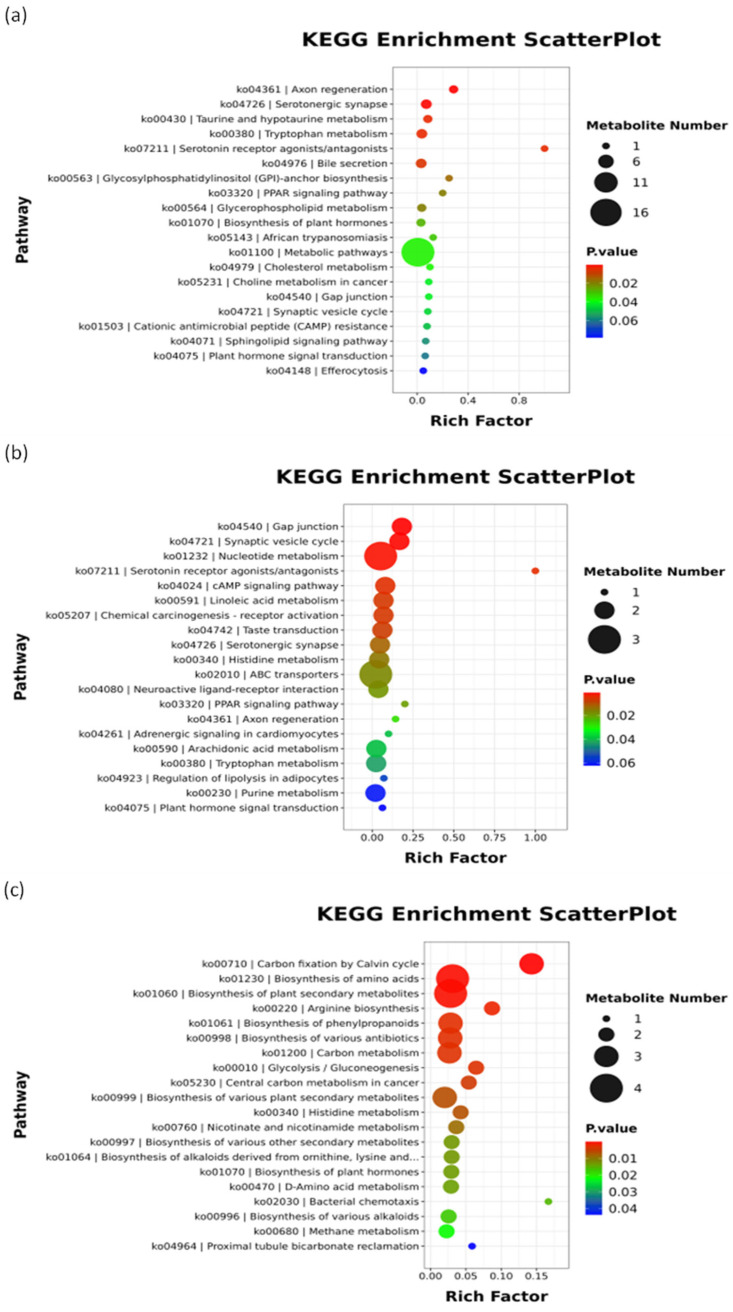
Bubble chart of enriched pathways for kidney metabolite differentials in *Micropterus salmoides* following oral gavage administration of 30 mg/kg OXO: (**a**) Pathway bubble chart: K-T vs. K-CON; (**b**) Pathway bubble chart: K-T vs. K-E; (**c**) Pathway bubble chart: K-E vs. K-CON.

A comparison between the OXO-administered group at the K-T and the K-CON group revealed that the key significantly differential metabolites in the kidney were primarily enriched in metabolic pathways and several organismal systems functions. Among these, the metabolic pathways mainly included Amino acid metabolism. Regarding organismal systems functions, the differences were notably enriched in the Endocrine system, specifically the Peroxisome proliferator-activated receptor (PPAR) signaling pathway, which senses lipid signals and regulates lipid metabolism, glucose metabolism, energy metabolism, and inflammatory responses (Figure 5a). Metabolomic profiling revealed substantial perturbations in renal metabolic pathways following OXO exposure. A suite of key metabolites was significantly altered (*p* < 0.05): serotonin, xanthurenic acid, jasmonic acid, guanosine, D-glucurono-6,3-lactone, and phenyllactic acid were upregulated, while L-tryptophan, O-phosphoethanolamine, spermidine, methylimidazoleacetic acid, oleanolic acid, and homogentisic acid were downregulated. Integrated analysis revealed that OXO triggered a coordinated renal response centered on amino acid metabolism. Key differential metabolites, including serotonin, phenyllactic acid, D-glucurono-6,3-lactone, and others, demonstrated significant correlations (*p* < 0.05) with antioxidant markers (ROS, SOD, CAT) [26,27]. The overall metabolic profile—characterized by the depletion of L-tryptophan and cytoprotective oleanolic acid, alongside the accumulation of xanthurenic acid and other intermediates—collectively indicates the induction of inflammatory and immune responses [28,29,30,31,32]. Notably, the observed tryptophan depletion suggests the potential development of an immunosuppressive microenvironment, likely mediated by IDO-driven immunometabolic reprogramming [33]. The upregulation of D-glucurono-6,3-lactone signifies active Phase II detoxification and excretion of OXO [34]. Although quinolones are known inducers of oxidative stress, the absence of a significant rise in ROS (*p* > 0.05), coupled with the upregulation of protective metabolites like guanosine [35,36], points to the mobilization of adaptive cellular defense mechanisms that mitigated overt oxidative damage in the kidney of *Micropterus salmoides* following OXO challenge.

Compared to the K-E group, renal metabolomic profiling of the K-T group indicated that significantly altered metabolites were predominantly enriched in lipid metabolism and amino acid metabolic pathways (Figure 5b). Major metabolites exhibiting significant upregulation included 13-HODE, 13-OxoODE, methylhistidine, imidazoleacetic acid, 11,12-DiHETrE, hepoxilin A3, serotonin, and xanthurenic acid (*p* < 0.05). Among these, 13-HODE, 13-OxoODE, 11,12-DiHETrE, and hepoxilin A3 are oxidized derivatives of polyunsaturated fatty acids (PUFAs) that function as specialized pro-resolving lipid mediators [37]. Specifically, 13-HODE and 13-OxoODE serve as endogenous antioxidants [38], whereas 11,12-DiHETrE and hepoxilin A3 demonstrate anti-inflammatory properties [39,40]. Their elevated levels at 8 h post-dosing suggest enhanced activation of renal lipid antioxidant pathways and active modulation of inflammatory processes. The increase in methylhistidine may reflect muscle proteolysis providing amino acids and energy to support immune responses, stress adaptation, and metabolic demands [41]. Additionally, serotonin and imidazoleacetic acid are established biomarkers of physiological stress [42,43]. It is noteworthy that none of these metabolites remained significantly altered in the K-E group relative to K-CON (*p* > 0.05), indicating that the compound-mediated inflammatory regulation and stress responses had returned to baseline during the elimination phase.

Comparative metabolomics between the K-E and K-CON groups showed that differentially abundant metabolites were mainly associated with metabolic pathways such as energy metabolism, carbohydrate metabolism, amino acid metabolism, and biosynthesis of secondary metabolites (Figure 5c). Significantly upregulated metabolites included epicatechin and phosphoenolpyruvic acid (*p* < 0.05), while L-aspartic acid, dihydroxyacetone phosphate, and ornithine were notably downregulated (*p* < 0.05). The elevation of epicatechin, an endogenous antioxidant, likely contributes to protection against antibiotic-induced oxidative injury in renal tissue [44,45]. This effect could also be associated with the restoration of ROS, MDA, and SOD levels to normal ranges in the K-E group. The concurrent increase in phosphoenolpyruvic acid—a critical gluconeogenic precursor—and decrease in dihydroxyacetone phosphate, a glycolytic intermediate, implies a metabolic reconfiguration shifting glycolytic flux toward gluconeogenesis instead of further catabolism or lipogenesis [46,47]. This reprogramming may represent an adaptive renal response to chemical stress, such as increased energy expenditure and detoxification load, intended to sustain glucose homeostasis and support ATP-demanding processes including the urea cycle. Antibiotic-induced cellular damage is likely accompanied by elevated ammonia and nitrogenous waste generation [48,49,50,51]. The reduced levels of L-aspartic acid and ornithine are consistent with accelerated urea cycle activity, wherein aspartate donates nitrogen for citrulline formation via argininosuccinate synthase, and ornithine is cyclically consumed in the citrulline-argininosuccinate loop, thereby promoting detoxification of ammonia into urea for excretion.

#### 3.3.3. Biochemical Parameters Differential Analysis of Kidney

Cytochrome P450 (CYP-ECOD) is named based on its unique absorption band at 450 nm when bound to carbon monoxide [52]. As one of the largest enzyme superfamilies in living organisms, Cytochrome P450 enzymes are responsible for approximately 80% of oxidative metabolism and mediate the metabolism of nearly 50% of commonly used drugs in humans [53], facilitating their elimination. It also plays critical roles in the detoxification and activation of environmental toxins [54,55], making it highly significant in reflecting drug metabolism dynamics. Excessively high CYP450 activity in target organs may accelerate drug metabolism, potentially reducing therapeutic efficacy, whereas excessively low activity could lead to drug accumulation and even toxicity.

Reactive oxygen species (ROS) are highly reactive molecules containing oxygen free radicals. ROS levels that exceed the capacity of the cellular antioxidant defense system can induce oxidative stress [56], which in turn may lead to cellular damage or even pathological changes in proteins, lipids, and DNA [57].

Malondialdehyde (MDA) content serves as a key indicator for assessing the extent of lipid peroxidation. When oxygen free radicals attack polyunsaturated fatty acids in biological membranes, they initiate lipid peroxidation, leading to the formation of lipid peroxides. As a cytotoxic end-product of this process, the level of MDA directly reflects the degree of oxidative damage to cellular lipids [58].

Superoxide dismutase (SOD) is a crucial antioxidant enzyme that protects cells from oxidative damage by catalyzing the dismutation of superoxide anion radicals (O_2_^−^) into less harmful hydrogen peroxide (H_2_O_2_) and oxygen (O_2_), thereby reducing cytotoxicity [59].

Catalase (CAT) reduces hydrogen peroxide levels by catalyzing its decomposition into water and oxygen. In coordination with other antioxidant enzymes such as superoxide dismutase and glutathione peroxidase, CAT forms a critical defense line against oxidative damage in cells [60].

A comparative analysis was conducted on the five biochemical indicators in the kidney of *Micropterus salmoides* following OXO administration by oral gavage, revealing significant differences. Kidney CYP450 enzyme activity was markedly induced, with both the K-T and K-E groups showing significantly higher levels than the K-CON group (*p* < 0.01; Figure 4(c1)). This suggests that OXO undergoes metabolic processing in the kidneys. Notably, the sustained high CYP-ECOD activity, observed even after the peak drug concentration was reached and during its decline, likely facilitated drug clearance in this primary accumulation organ, thereby reducing intra-renal accumulation and the potential for toxicity. Concurrently, both ROS and SOD levels were significantly elevated at the peak drug concentration time point (K-T) compared to the K-E and K-CON groups (*p* < 0.01; Figure 4(c3,c5)). This coordinated increase implies that the rise in ROS, triggered by the peak drug concentration at 8 h post-administration, activated a compensatory antioxidant response through SOD upregulation to neutralize free radicals and mitigate cellular damage. As the OXO concentration decreased, both ROS and SOD levels returned to baseline. Supporting the absence of overt oxidative damage, the MDA level in the K-T group was significantly lower than in both the K-E and K-CON groups (*p* < 0.01; Figure 4(c4)). This indicates not only a lack of lipid peroxidation but an apparent suppression of it at the peak of drug exposure. In contrast, renal CAT activity remained unchanged across all groups (*p* > 0.05; Figure 4(c2)).

Integrated analysis of the correlation heatmap (Figure 4d) and differential metabolite pathways in the kidney revealed a coordinated response to OXO. This response was characterized by significant alterations in key metabolites linked to amino acid metabolism. Based on this premise, a subset of major differential metabolites demonstrating significant links to amino acid metabolism was chosen to examine their correlations with antioxidant markers. Specifically, serotonin, D-glucurono-6,3-lactone, phenyllactic acid, and jasmonic acid showed significant correlations (*p* < 0.05) with the levels of ROS, SOD, and CAT. Meanwhile, homogentisic acid, spermidine, oleanolic acid, and L-tryptophan were significantly correlated (*p* < 0.05) with both ROS and SOD levels. Additionally, guanosine levels correlated significantly with ROS (*p* < 0.05), while imidazoleacetic acid correlated significantly with SOD (*p* < 0.05). The collective changes in these metabolites indicate the induction of an inflammatory/immune response in the kidney following OXO intake, alongside the activation of corresponding protective mechanisms.

#### 3.3.4. Metabolomic Differential Analysis of Muscle

**Figure 6:**
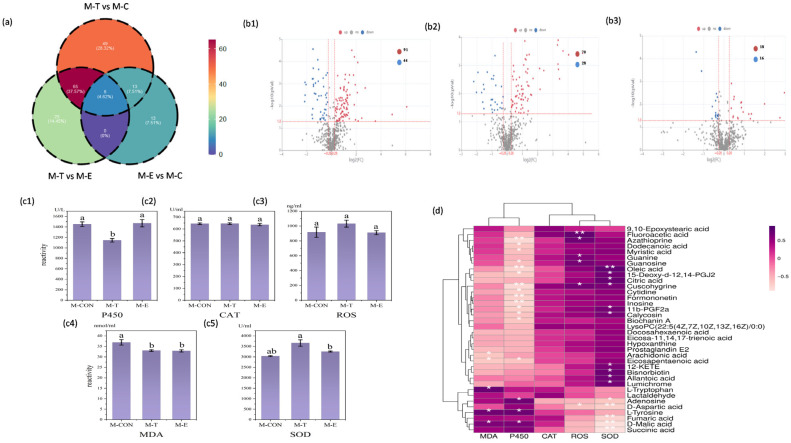
Muscle metabolic profiling and biochemical correlations in *Micropterus salmoides* following oral gavage administration of 30 mg/kg OXO: (**a**) Venn diagram of muscle metabolites; (**b1**) Volcano plot: M-T vs. M-CON; (**b2**) Volcano plot: M-T vs. M-E; (**b3**) Volcano plot: M-E vs. M-CON; (**c1**) P450 activity in muscle groups; (**c2**) CAT activity in muscle groups; (**c3**) ROS activity in muscle groups; (**c4**) MDA content in muscle groups; (**c5**) SOD activity in muscle groups; (**d**) Metabolite-biomarker correlation heatmap in muscle. Data are marked with asterisks to indicate significant differences: **, *p* < 0.01; *, a, b, *p* < 0.05.

**Figure 7:**
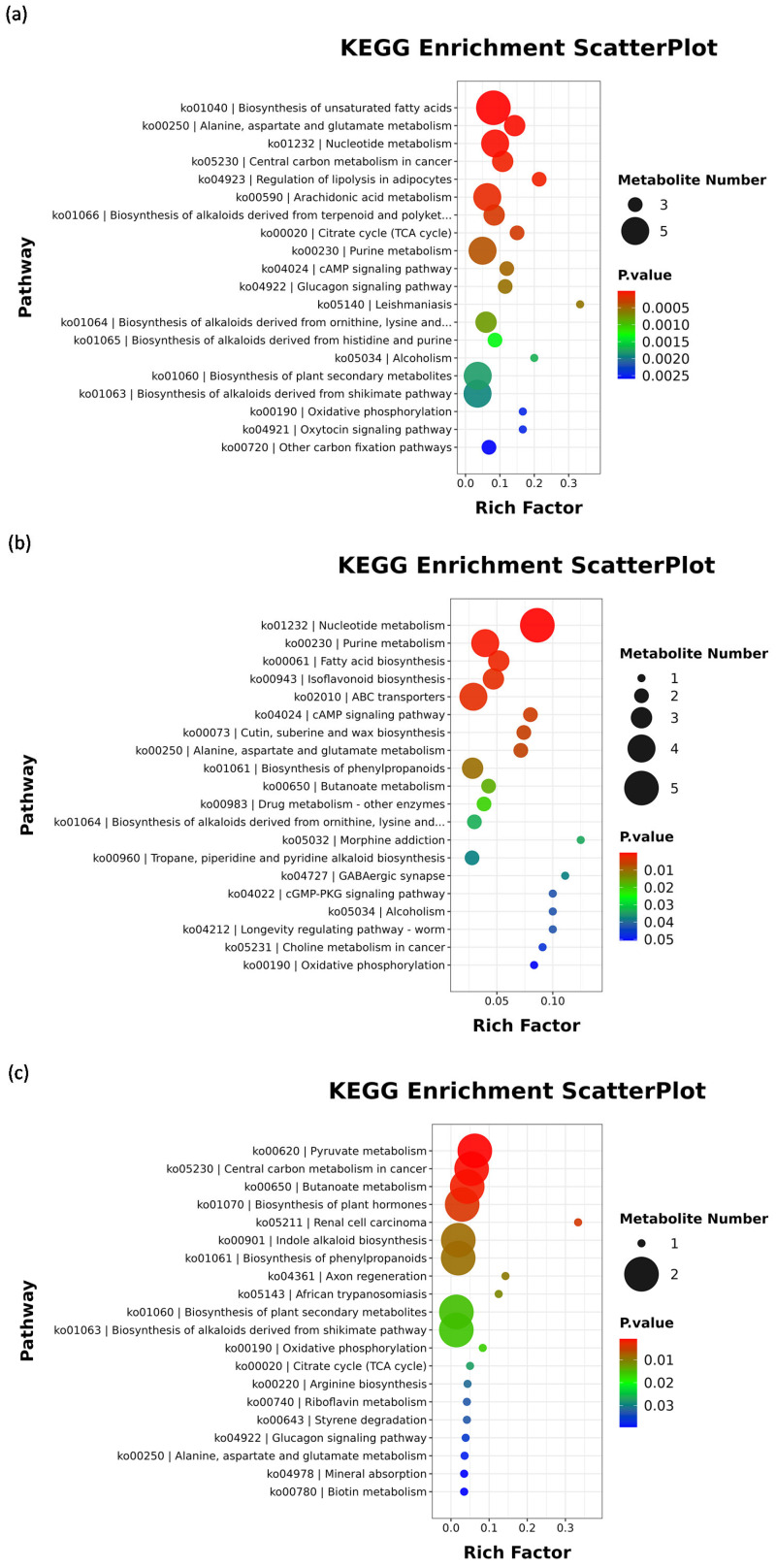
Bubble chart of enriched pathways for muscle metabolite differentials in *Micropterus salmoides* following oral gavage administration of 30 mg/kg OXO: (**a**) Pathway bubble chart: M-T vs. M-CON; (**b**) Pathway bubble chart: M-T vs. M-E; (**c**) Pathway bubble chart: M-E vs. M-CON.

This experiment identified key common differential metabolites by comparing the muscle metabolomes among the M-T, M-E, and M-CON following oral administration of OXO via medicated feed. The QC heatmap of muscle metabolomics, intra-group sample reproducibility, and PLS-DA evaluation of inter-group differences in metabolomics are provided in the Supplementary Materials (Figures S8 and S9). The statistics of muscle secondary metabolites across multiple comparison groups and the muscle differential metabolites from individual group comparisons are also available in the Supplementary Materials (Figures S10 and S11). A total of 135 differential metabolites were detected between the M-T and M-CON groups, among which 91 were significantly upregulated and 44 were significantly downregulated (Figure 6(b1)). Between the M-T and M-E groups, 98 differential metabolites were identified, with 70 upregulated and 28 downregulated (Figure 7b). Comparison of the M-E and M-CON groups revealed 34 differential metabolites, including 18 upregulated and 16 downregulated (Figure 6(b2)). Venn analysis was performed to identify common differential metabolites across comparison groups.

Metabolomic analysis revealed significant overlaps in metabolites across comparative groups (Figure 6a): 21 metabolites were common to both the M-T vs. M-CON and M-E vs. M-CON comparisons; 73 metabolites were shared between the M-T vs. M-CON and M-T vs. M-E comparisons; 8 metabolites overlapped between the M-E vs. M-CON and M-T vs. M-E comparisons; and 8 core metabolites were consistently identified across all three comparison sets (M-T vs. M-CON, M-E vs. M-CON, and M-T vs. M-E). These 8 shared metabolites were: OXO, 3-Hydroxyhexanoylcarnitine, D-Malic acid, Trachelanthine, Alfentanil, L, L-Cyclo(leucylprolyl), Ajmaline, and (R)-Cloxazolam.

#### 3.3.5. Functional Enrichment Analysis of Differential Metabolites of Muscle

Muscle KEGG enrichment analysis and metabolite correlation analysis are provided in the Supplementary Materials (Figures S12 and S13).

Compared to the M-CON group, the muscle tissue of M-T group exhibited significant alterations in metabolite profiles, primarily enriched in pathways associated with lipid metabolism, amino acid metabolism, nucleotide metabolism, energy metabolism, and carbohydrate metabolism (Figure 7a). Significantly upregulated metabolites (*p* < 0.05) included eicosa-11,14,17-trienoic acid, eicosapentaenoic acid (EPA), arachidonic acid (AA), docosahexaenoic acid (DHA), oleic acid, citric acid, prostaglandin E_2_ (PGE_2_), 11β-prostaglandin F_2_α (11β-PGF_2_α), 15-deoxy-Δ^12^,^14^-prostaglandin J_2_ (15d-PGJ_2_), 12-keto-eicosatetraenoic acid (12-KETE), inosine, guanosine, hypoxanthine, and allantoic acid. Conversely, metabolites such as succinic acid, fumaric acid, D-aspartic acid, L-tyrosine, and D-malic acid were significantly downregulated (*p* < 0.05). The elevated lipid mediators—including eicosa-11,14,17-trienoic acid, EPA, DHA, PGE_2_, AA, 11β-PGF_2_α, 15d-PGJ_2_, and 12-KETE—collectively indicate activated oxidative metabolism of membrane phospholipid-derived polyunsaturated fatty acids (PUFAs). OXO-induced oxidative stress likely initiated phospholipase-mediated hydrolysis, liberating precursor PUFAs such as arachidonic acid (AA, ω-6) and eicosapentaenoic acid (EPA, ω-3). Pro-inflammatory mediators derived from AA (PGE_2_, 11β-PGF_2_α, 12-KETE) promoted inflammatory responses [61,62,63], while anti-inflammatory and pro-resolving mediators from EPA [64,65,66] and 15d-PGJ_2_ (from AA) [67,68] facilitated inflammation resolution. This inflammatory process is energy-intensive, evidenced by increased citric acid supplying acetyl-CoA for biosynthesis, alongside ATP depletion activating purine degradation (accumulating inosine, hypoxanthine, allantoic acid). The concomitant rise in citric acid [69,70] and decline in TCA cycle intermediates (D-malic acid, succinic acid, fumaric acid) [71,72] suggest redirected carbon flux toward inflammatory lipid and nucleotide synthesis over energy production. Notably, accumulated purine degradation products impart bitterness, while extensive PUFA peroxidation causes oxidative rancidity. Elevated citric acid may also contribute sourness. Thus, at peak OXO concentration, the flavor quality of *Micropterus salmoides* muscle is substantially compromised, rendering it organoleptically unacceptable.

Comparative analysis of differential metabolites between the M-T and M-E groups indicated that the alterations were primarily associated with nucleotide metabolism, lipid metabolism, amino acid metabolism, xenobiotics biodegradation and metabolism, carbohydrate metabolism, and the biosynthesis of other secondary metabolites (Figure 7b). Significantly upregulated metabolites included: inosine, guanosine, cytidine, guanine, dodecanoic acid, myristic acid, oleic acid, biochanin A, calycosin, formononetin, 9,10-epoxystearic acid, azathioprine, fluoroacetic acid, cuscohygrine, trigonelline, and LysoPC(22:5(4Z,7Z,10Z,13Z,16Z)/0:0) (*p* < 0.05). In contrast, adenosine and succinic acid were significantly downregulated (*p* < 0.05). Consistent with the comparison between M-T and M-CON groups, nucleotide metabolites (inosine, guanosine, cytidine, guanine), free fatty acids (dodecanoic acid, myristic acid, oleic acid), and lysophosphatidylcholine (LysoPC) remained markedly elevated during the peak-exposure period, reflecting sustained enhancement of cellular metabolic turnover. Concurrently, xenobiotic compounds were biotransformed into metabolites such as azathioprine, fluoroacetic acid, cuscohygrine, and trigonelline. The accumulation of phytoestrogens (e.g., biochanin A, calycosin, formononetin) and alkaloids (e.g., cuscohygrine, trigonelline) suggests direct or indirect inhibition of the hepatic detoxification system—particularly the cytochrome P450 (CYP) enzyme family—by the antibiotic exposure. This suppression likely compromised the liver’s ability to metabolize and clear exogenous and plant-derived compounds, resulting in their tissue deposition [73,74]. These findings are consistent with documented inhibitory effects of antibiotics on drug-metabolizing enzymes and are further corroborated by the significantly reduced activity of the muscle CYP system in the M-T group compared to both M-CON and M-E groups. In contrast, the upregulation of adenosine and succinic acid in the elimination group (M-E) may reflect mitochondrial dysfunction or feedback inhibition arising from reduced energy demand and dysregulated metabolic flux [75,76]. This could disrupt adenosine recycling for ATP resynthesis and lead to a bottleneck in the TCA cycle at the succinate dehydrogenase step, impairing complete oxidative metabolism [77].

To elucidate the metabolic status during the elimination phase, a comparative metabolomic analysis was conducted between the M-E and M-CON groups. The results demonstrated that significantly altered metabolites were primarily enriched in pathways associated with carbohydrate metabolism and amino acid metabolism (Figure 7c). Notably, lumichrome and bisnorbiotin were significantly upregulated (*p* < 0.05), whereas fumaric acid, lactaldehyde, D-malic acid, and L-tryptophan were significantly downregulated (*p* < 0.05). The decrease in fumaric acid and D-malic acid—key intermediates in the tricarboxylic acid (TCA) cycle—suggests a reduced flux through the TCA cycle or a partial interruption of mitochondrial oxidative metabolism. This impairment is likely to diminish the supply of reducing equivalents (NADH and FADH_2_) to the electron transport chain, ultimately resulting in compromised ATP synthesis. The downregulation of L-tryptophan may reflect enhanced protein catabolism and immune activation under energy deficit conditions, leading to depletion of amino acid pools. The reduction in lactaldehyde further supports the overall downregulation and reprogramming of energy metabolic pathways [78]. The accumulation of lumichrome—a photodegradation product of riboflavin (vitamin B_2_)—implies high consumption of riboflavin for coenzyme synthesis (e.g., FAD), which is required by antioxidant enzymes such as glutathione reductase under oxidative stress. This accelerated riboflavin catabolism promotes lumichrome generation [79]. Similarly, increased bisnorbiotin, a biotin (vitamin B_7_) derivative, indicates disrupted vitamin metabolism likely due to metabolic stress [80]. Both metabolites reflect abnormalities in vitamin homeostasis. However, integrated analysis with physiological parameters showed that the activities of SOD and CAT in muscle tissue did not differ significantly from the control (*p* > 0.05), while MDA levels were significantly lower than those in the control group (*p* < 0.05). Moreover, no significant differences were observed between the M-E and M-CON groups in lipid metabolism, nucleotide metabolism, or in metabolites such as adenosine and succinic acid (*p* > 0.05). Thus, it can be inferred that after OXO clearance, no significant oxidative stress or mitochondrial dysfunction persisted in muscle tissue. Furthermore, metabolites that were significantly altered in the M-T versus M-CON comparison—including inosine, guanosine, hypoxanthine, and allantoic acid—exhibited no significant differences between the M-E and M-CON groups (*p* > 0.05). This indicates that upon drug elimination, muscle metabolite levels related to flavor attributes returned to baseline, suggesting the absence of persistent adverse effects on sensory quality.

#### 3.3.6. Biochemical Parameters Differential Analysis of Muscle

Analysis of biochemical indicators in the muscle of *Micropterus salmoides* after OXO exposure revealed distinct responses. CYP450 activity in the M-T group was significantly lower than that in the M-E and M-CON groups (*p* < 0.01; Figure 6(c1)). Given the concomitantly low drug concentration at the peak time point, this suggests limited accumulation and metabolic impact of OXO in muscle tissue. In contrast to the renal profile, ROS levels in the muscle showed no significant differences across time points (*p* > 0.05; Figure 6(c2)). However, SOD activity in the M-T group was significantly higher than in the M-E and M-CON groups (*p* < 0.01; Figure 6(c3)). This suggests that, analogous to the mechanism in the kidney, a compensatory upregulation of SOD activity was triggered to efficiently scavenge free radicals, thereby maintaining ROS at baseline levels. Furthermore, MDA levels in both the M-T and M-E groups were significantly lower than those in the M-CON group (*p* < 0.01; Figure 6(c4)). Integrated with the analysis of differential metabolites and enriched pathways in muscle, these findings collectively indicate that although OXO intake provoked antioxidant stress and the mobilization of polyunsaturated fatty acids, it did not culminate in significant peroxidative damage. No significant differences in CAT activity were detected in the muscle across all groups (*p* > 0.05; Figure 6(c5)).

Integrated analysis of the correlation heatmap between muscle biochemical indicators and metabolites (Figure 6d), along with pathway enrichment analysis of differential metabolites, was focused on correlations pertaining to lipid metabolism and oxidative stress. The results revealed that multiple lipid metabolism-related metabolites, including succinic acid, D-malic acid, fumaric acid, D-aspartic acid, adenosine, lumichrome, bisnorbiotin, allantoic acid, 12-KETE, calycosin, 11b-PGF2a, cuscohygrine, citric acid, 15-deoxy-Δ-12,14-PGJ2, and oleic acid, showed significant correlations with SOD levels (*p* < 0.05). Additionally, metabolites such as D-aspartic acid, cuscohygrine, guanosine, fluoroacetic acid, and azathioprine were significantly correlated with ROS levels (*p* < 0.05). Furthermore, numerous differential metabolites exhibited significant negative correlations with CYP450 activity (*p* < 0.05). Based on these findings, it is hypothesized that the oxidative stress induced by OXO triggers the production of specific pro-resolving mediators. The robust inflammatory and immune responses observed in the muscle are interpreted as an adaptive mechanism that facilitates healthy metabolic regulation and tissue homeostasis. 

## 4. Conclusions

This study demonstrates that OXO exhibits significant inhibitory effects against *Aeromonas hydrophila* NJ-35 in vitro. A single oral dose of 30 mg/kg OXO in *Micropterus salmoides* shows tissue-specific distribution and metabolic characteristics. The kidney was identified as the primary site of metabolism, reaching its peak concentration (Tmax) at 8 h post-administration, while the muscle, as the major edible tissue, showed a later Tmax at 24 h. Based on pharmacokinetic data, a withdrawal period of 17 days (408 h) is recommended. To further elucidate the physiological mechanisms, kidney and muscle tissues were collected at their respective peak-concentration times (8 h and 24 h) and during the elimination phase (72 h) for metabolomic and physio-biochemical analyses. The results revealed that although OXO exposure induced disturbances in amino acid metabolism in the kidney and altered lipid metabolism in the muscle, no irreversible oxidative damage was observed. The significant reduction in differential metabolites post-elimination confirms that adhering to the recommended withdrawal period ensures edible safety. In conclusion, by integrating evidence from in vitro antibacterial tests, pharmacokinetics, and metabolomics, this study supports the safe use of OXO for controlling *Aeromonas hydrophila* infection *Micropterus salmoides* under the prescribed withdrawal period. Future investigations should address chronic exposure, environmental metabolite identification, and potential ecological impacts to ensure a comprehensive risk assessment.

## Figures and Tables

**Table 1:** Pharmacokinetic parameters of OXO in *Micropterus salmoides* following oral gavage.

Pharmacokinetic Parameters	Unit	Brain	Liver	Intestine	Kidney	Muscle	Plasma
A	g/L	22.24	164.07	59.20	17.87	29.26	2.84
α	1/h	0.07	0.16	0.09	0.03	0.15	0.01
B	g/L	0.64	119.08	0.29	0.47	26.51	0.01
β	1/h	0.05	0.12	0.01	0.01	0.10	0.01
Ka	1/h	0.09	0.28	0.13	0.15	15,152.38	0.098
t1/2α	h	9.38	4.45	7.45	20.14	4.59	49.23
t1/2β	h	69.32	6.05	69.32	69.32	7.21	69.32
K10	1/h	0.02	0.91	0.05	0.03	0.65	0.01
K12	1/h	0.04	0.77	0.04	0.01	0.53	0.01
K21	1/h	0.02	0.14	0.01	0.01	0.12	0.01
Tmax	h	6	12	8	8	24	48
Cmax	mg/L	3.89	6.42	10.64	17.99	4.54	6.22
AUC (0–∞)	mg/L*h	161.20	157.30	326.81	477.99	85.84	188.01

Note: A: Distribution phase coefficient; α: Distribution phase rate constant; B: Elimination term coefficient; β: Elimination phase rate constant; Ka: Absorption rate constant; t_1_/_2_α: Distribution half-life; t_1_/_2_β: Elimination half-life; K_10_: Central compartment elimination rate constant; K_12_: Central-to-peripheral transfer rate constant; K_21_: Peripheral-to-central transfer rate constant; T_max_: Time to top concentration; C_max_: Maximum concentration; AUC: Area under the curve.

## Data Availability

Due to privacy considerations, the pharmacokinetic and biochemical data from this study are not publicly available. However, these data can be made accessible to readers upon reasonable request to the corresponding author and after signing a data use agreement. The raw metabolomics data generated in this study have been deposited in the National Genomics Data Center, China National Center for Bioinformation, under the BioProject accession number PRJCA051728. All other data are included in the Supplementary Materials.

## Supplementary Materials

The following supporting information can be downloaded at: https://www.mdpi.com/article/10.3390/antiox15030283/s1, Section S1. Pharmacokinetic experimental liquid chromatography parameters description; Section S2. Metabolomics experimental liquid chromatography parameters description; Section S3. Mass spectrometry parameters description; Section S4. Information analysis description; Figure S1. LC-MS Ion Chromatogram; Figure S2. Kidney metabolomics QC heatmap; Figure S3. PLS-DA evaluation of intra-group sample reproducibility and inter-group differences in kidney metabolomics.; Figure S4. Statistical bar chart of secondary metabolites in kidney across multiple comparison groups; Figure S5. Heatmap of kidney differential metabolites for single-group comparison; Figure S6. KEGG enrichment analysis of the kidney; Figure S7. Kidney metabolite correlation analysis; Figure S8. Muscle metabolomics QC heatmap; Figure S9. PLS-DA evaluation of intra-group sample reproducibility and inter-group differences in muscle metabolomics.; Figure S10. Statistical bar chart of secondary metabolites in muscle across multiple comparison groups; Figure S11. Heatmap of muscle differential metabolites for single-group comparison; Figure S12. KEGG enrichment analysis of the muscle; Figure S13. Muscle metabolite correlation analysis; Table S1. Formulas for Calculating Pharmacokinetic Parameters Beyond Observed Concentration-Time Curve Fitting; Table S2. Method recovery rate of OXO in various tissues of Micropterus salmoides.; Table S3. XCMS main parameters; Table S4. MetaX main parameters.
