# Supplementary material for: Integrative Pharmacokinetic and Metabolomic Analyses Reveal the Underlying Mechanisms of Metabolic Regulation and Support the Safe Use of Oxolinic Acid in Micropterus salmoides
Source: Antioxidants (Basel). 2026 Feb 25;15(3):283. doi: 10.3390/antiox15030283 (PMC13024334; doi:10.3390/antiox15030283)
Supplement: Supplementary file 1 [file antioxidants-15-00283-s001.zip › antioxidants-4110737-Supplementary.pdf]

Integrative Pharmacokinetic and Metabolomic Analyses Reveal the Underlying Mechanisms of Metabolic Regulation and Support the Safe Use of Oxolinic Acid in *Micropterus salmoides*.

Jiayin Yang[a,b], Mingxiao Li[c], Xi Chen[b], Chao Song[b,c], Linmin Fan[b,c], Liping Qiu[b], Dandan Li[b],Huimin Xu[b], Xiyan Mu[*], Tiejun Li[*], Shunlong Meng[a,b,c,]**

[a] College of Fisheries and Life Science, Shanghai Ocean University, Shanghai 200120, China

[b] *Freshwater Fisheries Research Center, Chinese Academy of Fishery Sciences, 214081 Wuxi, China*

[c] *Wuxi Fisheries College, Nanjing Agricultural University, 214081 Wuxi, China*

[d] *Zhejiang Marine Fisheries Research Institute, 316021, Zhoushan, China*

[*] Corresponding author.

[**] Corresponding author at: *Freshwater Fisheries Research Center, Chinese Academy of Fishery Sciences, 214081 Wuxi, China*

E-mail addresses: muxiyan@caas.cn(XY. Mu); litiejun1982@126.com(TJ. Li) mengsl@ffrc.cn (SL. Meng)

Section S1. Pharmacokinetic experimental liquid chromatography parameters description
Section S2. Metabolomics experimental liquid chromatography parameters description
Section S3. Mass spectrometry parameters description
Section S4. Information analysis description
Fig S1. LC-MS Ion Chromatogram
Fig S2. Kidney metabolomics QC heatmap
Fig S3. PLS-DA evaluation of intra-group sample reproducibility and inter-group differences in kidney metabolomics.
Fig S4. Statistical bar chart of secondary metabolites in kidney across multiple comparison groups
Fig S5. Heatmap of kidney differential metabolites for single-group comparison
Fig S6. KEGG enrichment analysis of the kidney
Fig S7. Kidney metabolite correlation analysis
Fig S8. Muscle metabolomics QC heatmap
Fig S9. PLS-DA evaluation of intra-group sample reproducibility and inter-group differences in muscle metabolomics.
Fig S10. Statistical bar chart of secondary metabolites in muscle across multiple comparison groups
Fig S11. Heatmap of muscle differential metabolites for single-group comparison
Fig S12. KEGG enrichment analysis of the muscle
Fig S13. Muscle metabolite correlation analysis
Table S1. Formulas for Calculating Pharmacokinetic Parameters Beyond Observed Concentration-Time Curve Fitting
Table S2. Method recovery rate of OXO in various tissues of Micropterus salmoides.
Table S3. XCMS main parameters
Table S4. MetaX main parameters

Section S1. Pharmacokinetic experimental liquid chromatography parameters description

Analysis was performed using an ultra-performance liquid chromatography-tandem mass spectrometry (UPLC-MS/MS) system (Waters, Massachusetts, USA). Liquid chromatography conditions: Column: ACQUITY UPLC BEH C18 (2.1 mm × 100 mm, 1.7 μm), Mobile phase A: 0.1% formic acid in water; Mobile phase B: acetonitrile, Flow rate: 0.3 mL/min, Column temperature: 40 °C, Injection volume: 3 μL, Gradient elution program: 0–1.0 min: 90% A, 1.0–8.0 min: 90% → 0% A, 8.0–10.0 min: 0% A, 10.0–10.1 min: 0% → 90% A.

Section S2. Metabolomics experimental liquid chromatography parameters description

All samples were acquired by the LC-MS system followed machine orders. Firstly, all chromatographic separations were performed using an ACQUITY UPLC system (Waters, Milford, MA, USA). An ACQUITY UPLC T3 column (100mm*2.1mm, 1.8μm, Waters, Milford, USA) was used for the reversed phase separation. The column oven was maintained at 40°C. The flow rate was 0.3 ml/min and the mobile phase consisted of solvent A (water, 5mM ammonium acetate and 5mM acetic acid) and solvent B (Acetonitrile). Gradient elution conditions were set as follows: 0-0.8 min, 2% B; 0.8-2.8 min, 2% → 70% B; 2.8-5.6 min, 70%→90% B; 5.6-6.4 min, 90%→100% B; 6.4–8.0 min, 100% B; 8.0-8.1 min, 100%→2% B; 8.1-10 min, 2% B.

Section S3. Mass spectrometry parameters description

TripleTOF 6600： A high-resolution tandem mass spectrometer TripleTOF 6600 (SCIEX, Framingham, MA, USA) was used to detect metabolites eluted from the column. The Q-TOF was operated in both positive and negative ion modes. The curtain gas was set 30 PSI, Ion source gas1 was set 60 PSI, Ion source gas2 was set 60 PSI, and an interface heater temperature was 500 ℃. For positive ion mode, the Ionspray voltage floating were set at 5000 V, respectively. For negative ion mode, the Ionspray voltage floating were set at -4500V, respectively. The mass spectrometry data were acquired in IDA mode. The TOF mass range was from 60 to 1200 Da. The survey scans were acquired in 150 ms and as many as 12 product ion scans were collected if exceeding a threshold of 100 counts per second (counts/s) and with a 1+ charge-state. Dynamic exclusion was set for 4 s. During the acquisition, the mass accuracy was calibrated every 20 samples. Furthermore, in order to evaluate the stability of the LC-MS during the whole acquisition, a quality control sample (Pool of all samples) was acquired after every 10 samples.

Section S4. Information analysis description

The acquired MS data pretreatments including peak picking, peak grouping, retention time correction, second peak grouping, and annotation of isotopes and adducts was performed using XCMS software. LC−MS raw data files were converted into mzXML format and then processed by the XCMS, CAMERA and metaX toolbox implemented with the R software. Each ion was identified by combining retention time (RT) and m/z data. Intensities of each peaks were recorded and a three

dimensional matrix containing arbitrarily assigned peak indices (retention time-m/z pairs), sample names (observations) and ion intensity information (variables) was generated. The online KEGG, HMDB database was used to annotate the metabolites by matching the exact molecular mass data (m/z) of samples with those from database. If a mass difference between observed and the database value was less than 10 ppm, the metabolite would be annotated and the molecular formula of metabolites would further be identified and validated by the isotopic distribution measurements. We also used a in-house fragment spectrum library of metabolites to validate the metabolite identidification. The intensity of peak data was further preprocessed by metaX. Those features that were detected in less than 50% of QC samples or 80% of biological samples were removed, the remaining peaks with missing values were imputed with the k-nearest neighbor algorithm to further improve the data quality. PCA was performed for outlier detection and batch effects evaluation using the pre-processed dataset. Quality control-based robust LOESS signal correction was fitted to the QC data with respect to the order of injection to minimize signal intensity drift over time. In addition, the relative standard deviations of the metabolic features were calculated across all QC samples, and those > 30% were then removed. Student t-tests were conducted to detect differences in metabolite concentrations between 2 phenotype.The P value was adjusted for multiple tests using an FDR (Benjamini–Hochberg). Supervised PLS-DA was conducted through metaX to discriminate the different variables between groups. The VIP value was calculated. A VIP cut-off value of 1.0 was used to select important features.

(a)

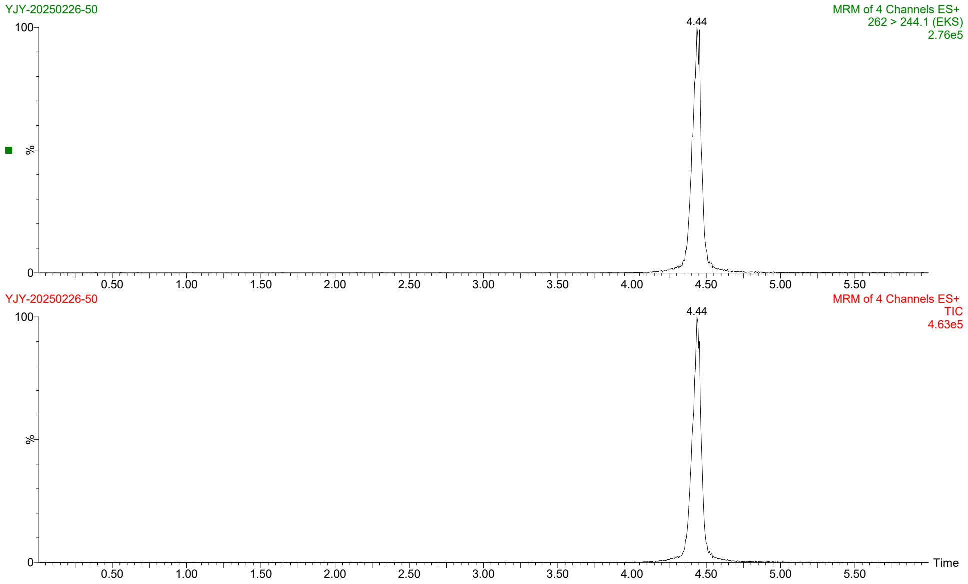

(b)

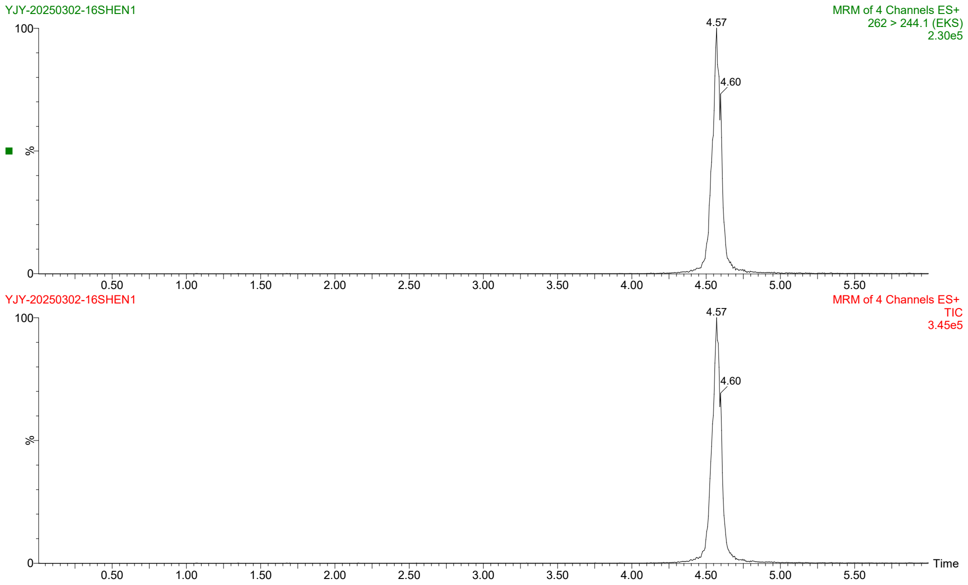

Fig S1. LC-MS Ion Chromatogram

a. OXO Standard LC-MS Ion Chromatogram

b. LC-MS Ion Chromatogram of OXO in *Micropterus salmoides* Tissues

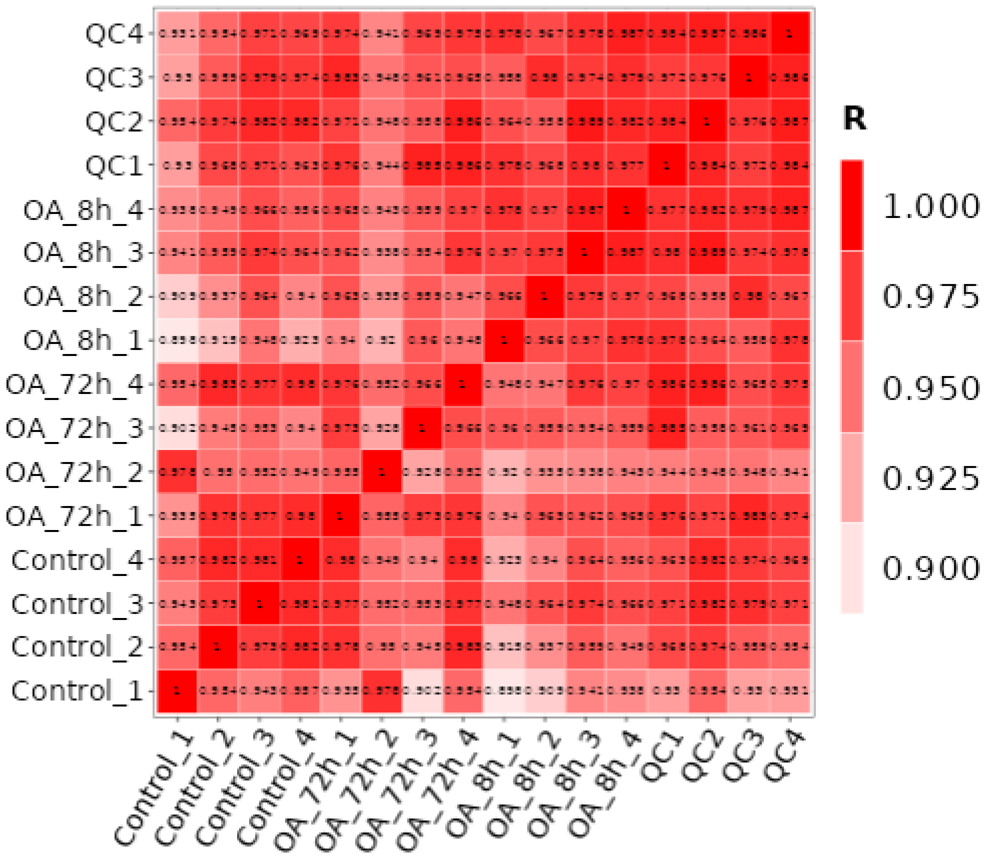

Fig S2. Kidney metabolomics QC heatmap

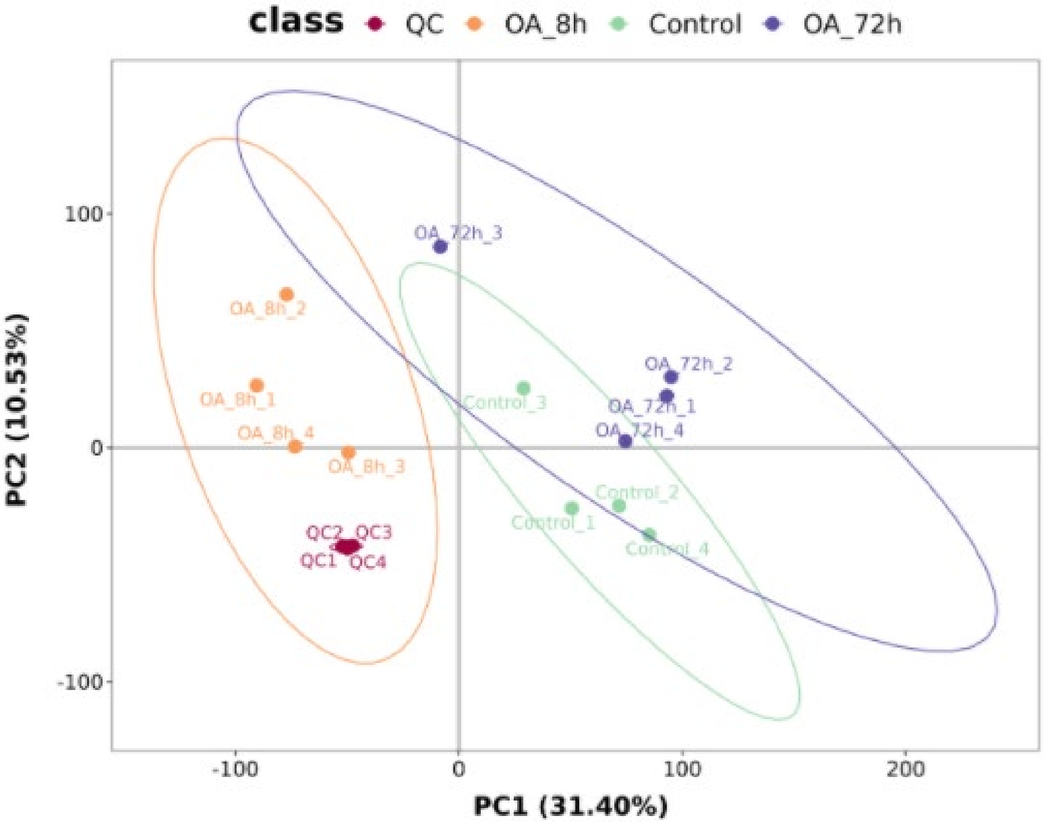

Fig S3. PLS-DA evaluation of intra-group sample reproducibility and inter-group differences in kidney metabolomics.

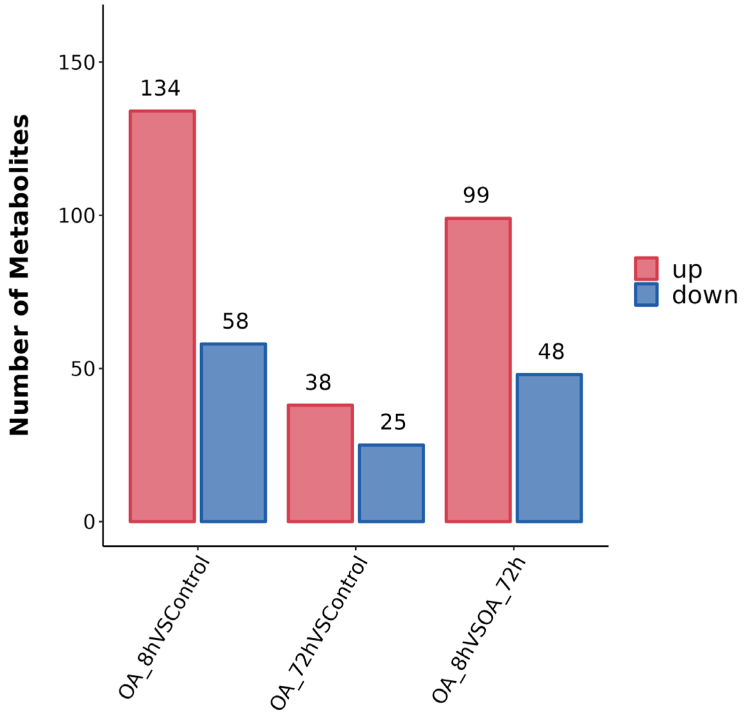

Fig S4. Statistical bar chart of secondary metabolites in kidney across multiple comparison groups

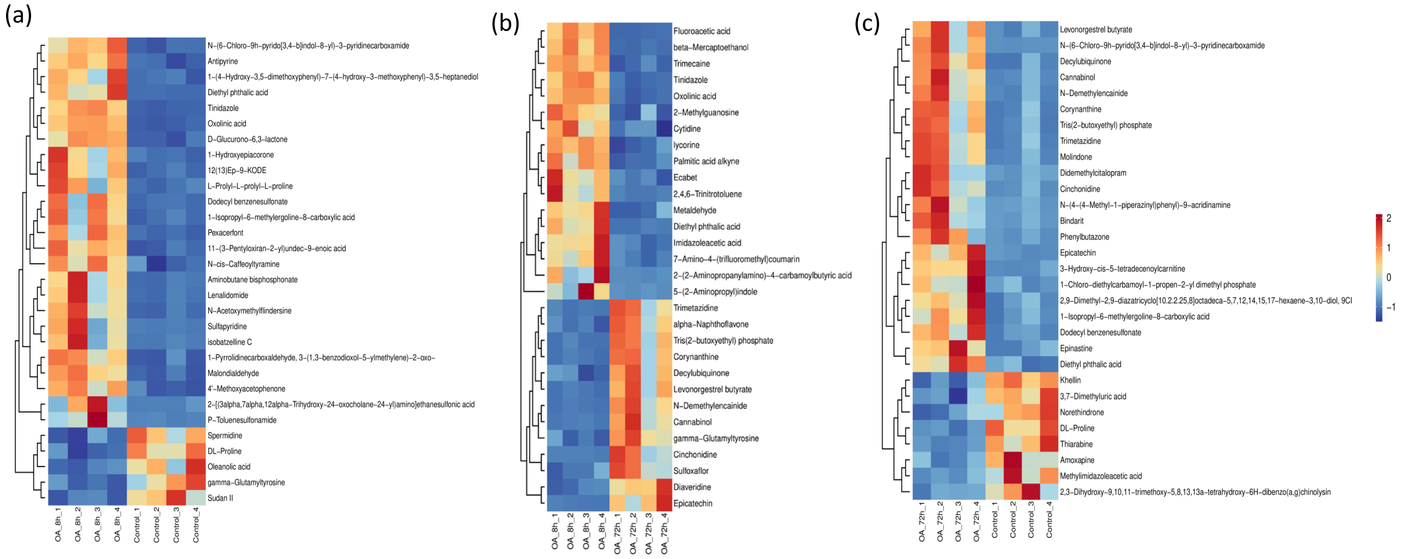

Fig S5. Heatmap of kidney differential metabolites for single-group comparison

a. Heatmap for single-group comparison (8h vs 72h)

b. Heatmap for single-group comparison (8h vs CON)

c. Heatmap for single-group comparison (72h vs CON)

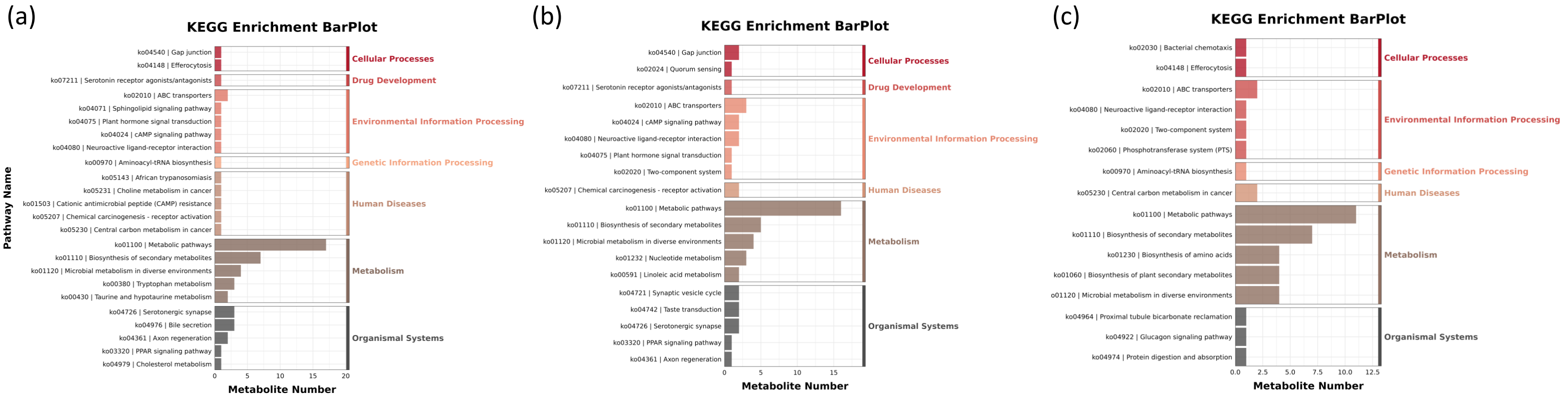

Fig S6. KEGG enrichment analysis of the kidney

a. KEGG enrichment analysis of the kidney(8h vs CON)

b. KEGG enrichment analysis of the kidney(8h vs 72h)

c. KEGG enrichment analysis of the kidney(72h vs CON)

(a)

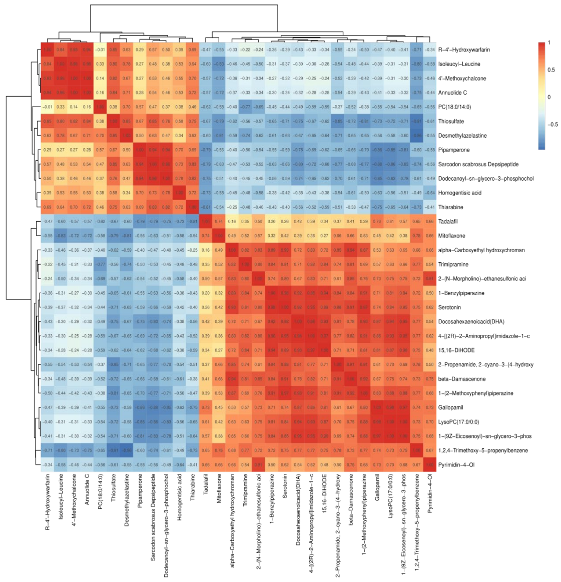

(b)

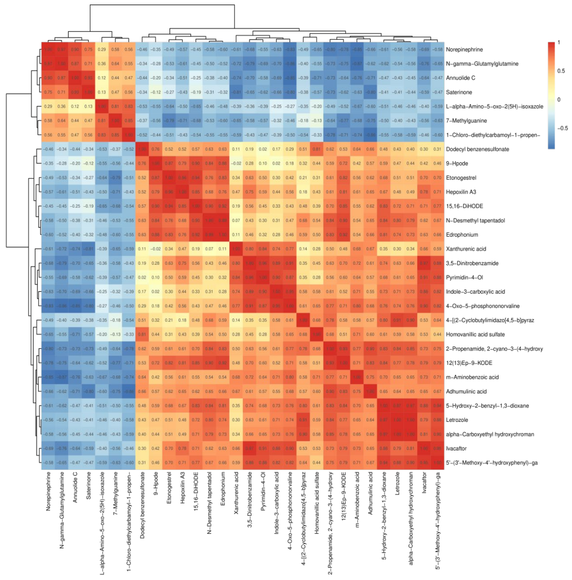

(c)

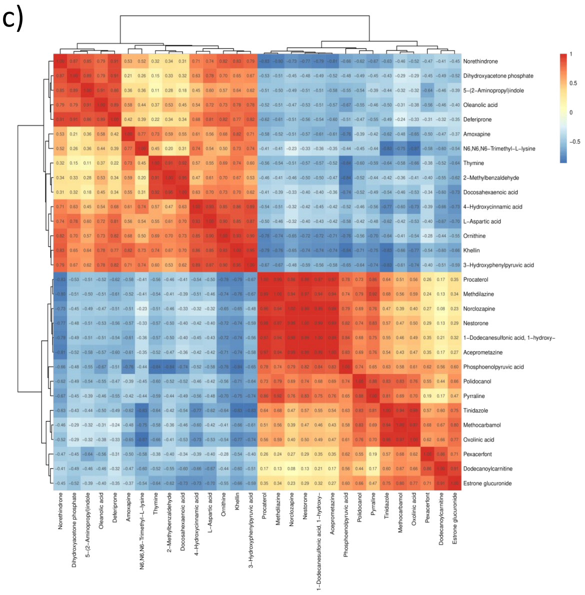

Fig S7. Kidney metabolite correlation analysis

1. Kidney metabolite correlation analysis(8h vs CON)

2. Kidney metabolite correlation analysis(8h vs 72h)

3. Kidney metabolite correlation analysis(72h vs CON)

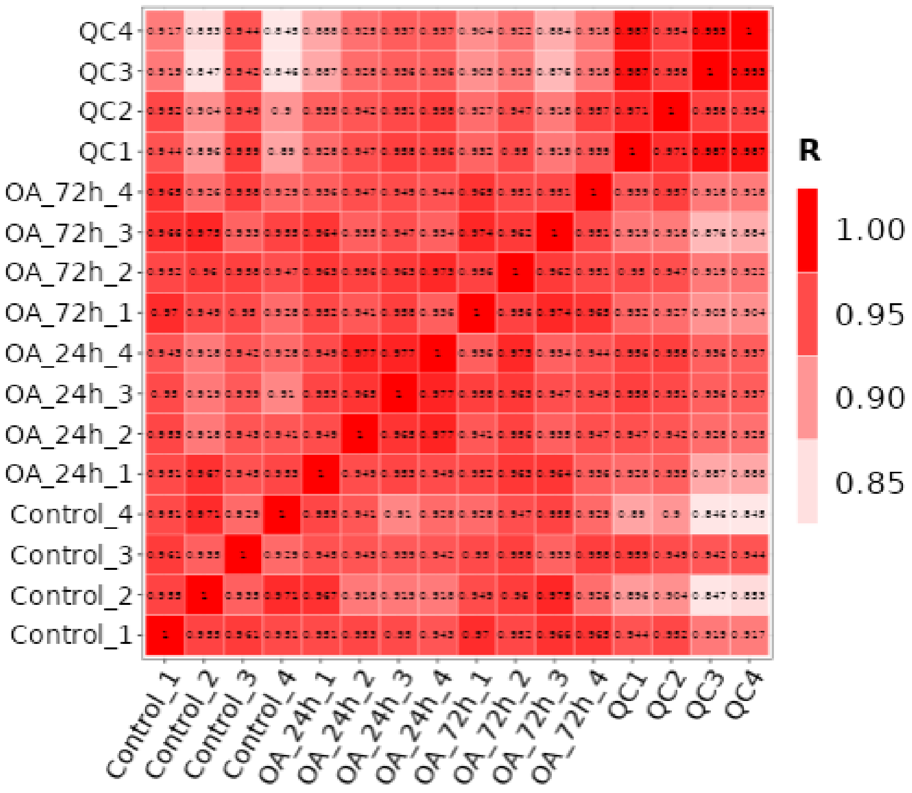

Fig S8. Muscle metabolomics QC heatmap

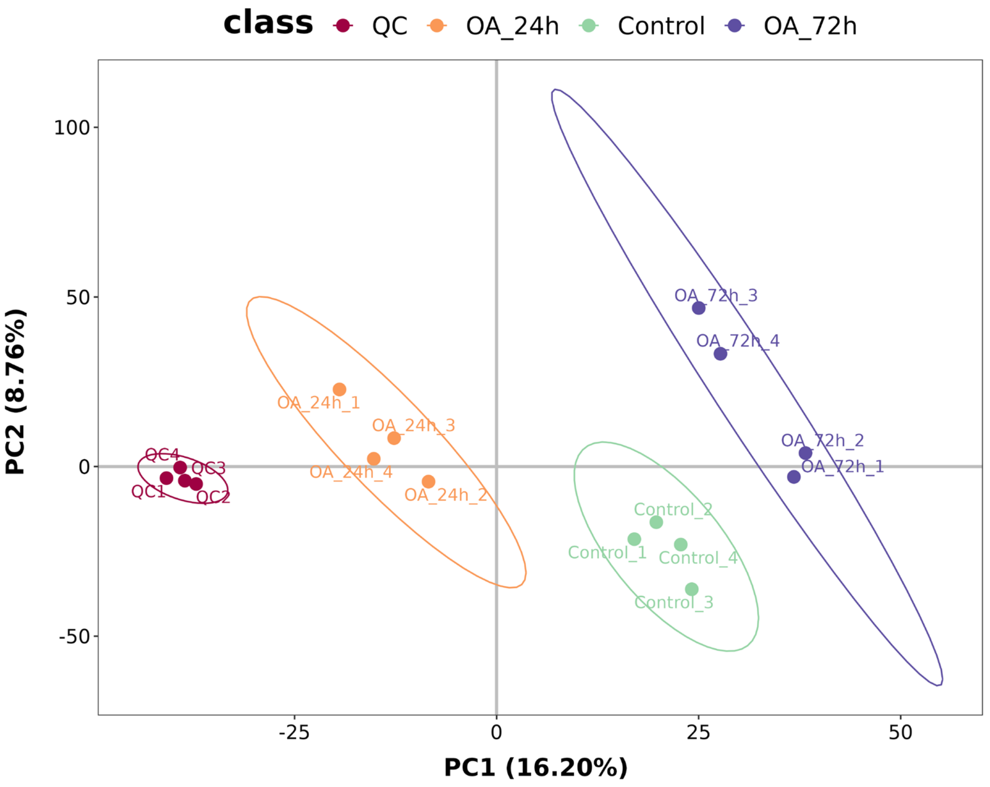

Fig S9. PLS-DA evaluation of intra-group sample reproducibility and inter-group differences in muscle metabolomics

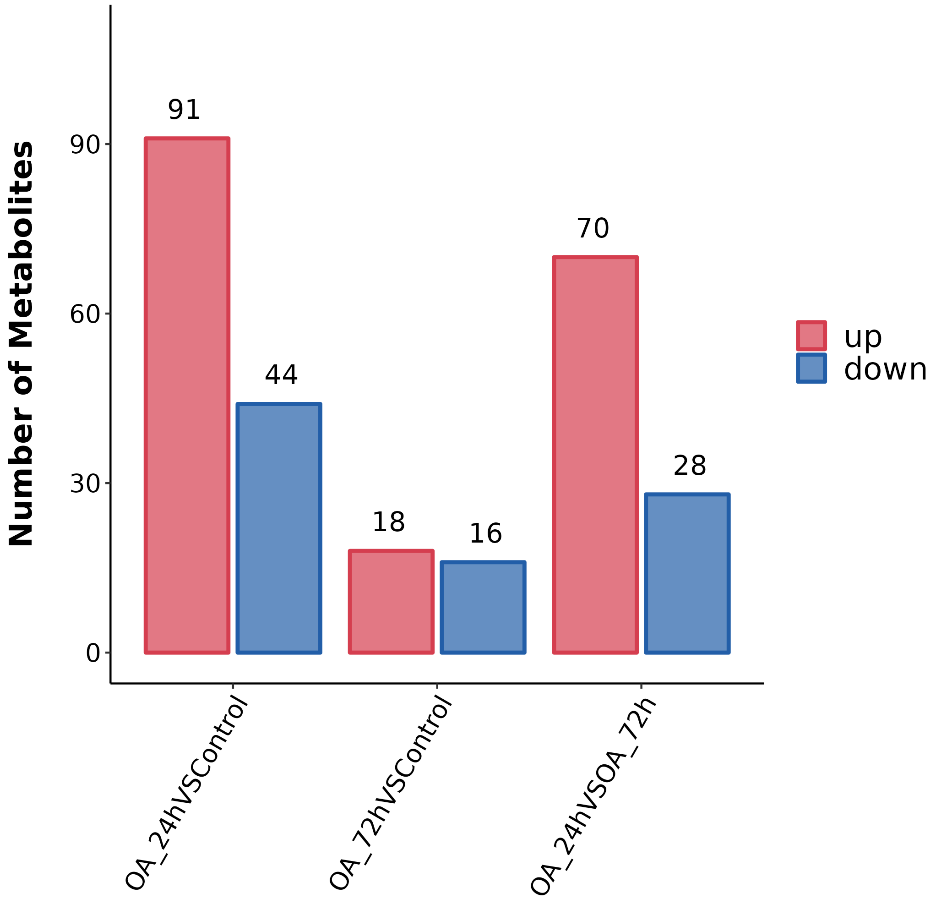

Fig S10. Statistical bar chart of secondary metabolites in muscle across multiple comparison groups

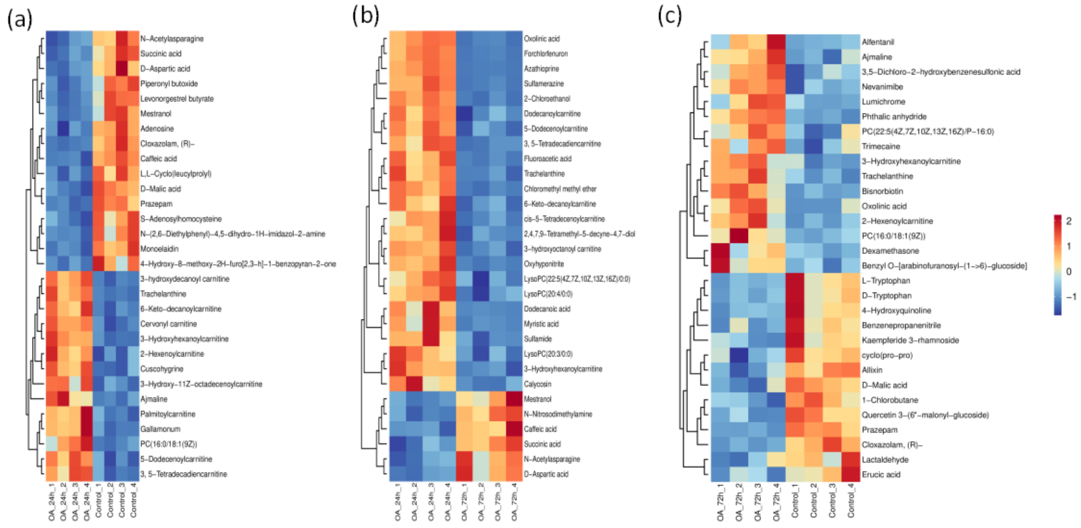

Fig S11. Heatmap of muscle differential metabolites for single-group comparison

a. **Heatmap for single-group comparison (24h vs CON)**

b. **Heatmap for single-group comparison (24h vs 72h)**

c. **Heatmap for single-group comparison (72h vs CON)**

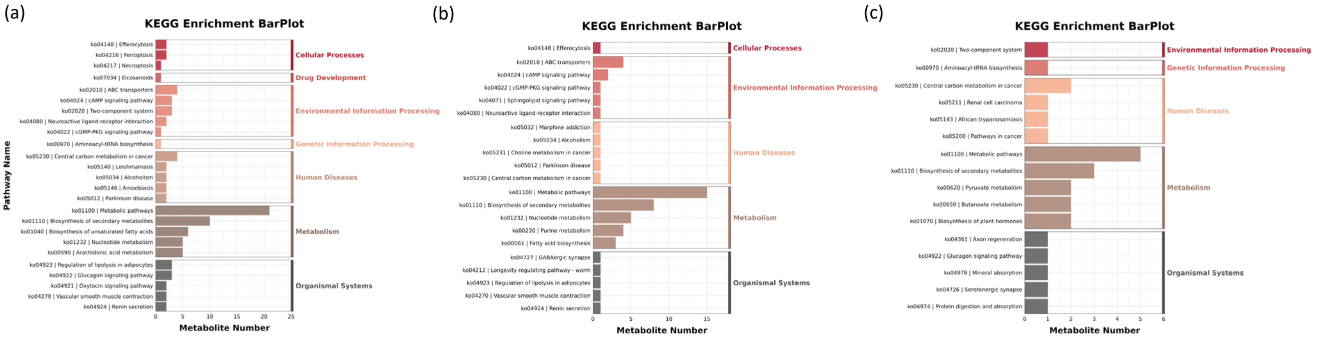

Fig S12. KEGG enrichment analysis of the muscle

a. KEGG enrichment analysis of the muscle (24h vs CON)

b. KEGG enrichment analysis of the kidney(24h vs 72h)

c. KEGG enrichment analysis of the kidney(72h vs CON)

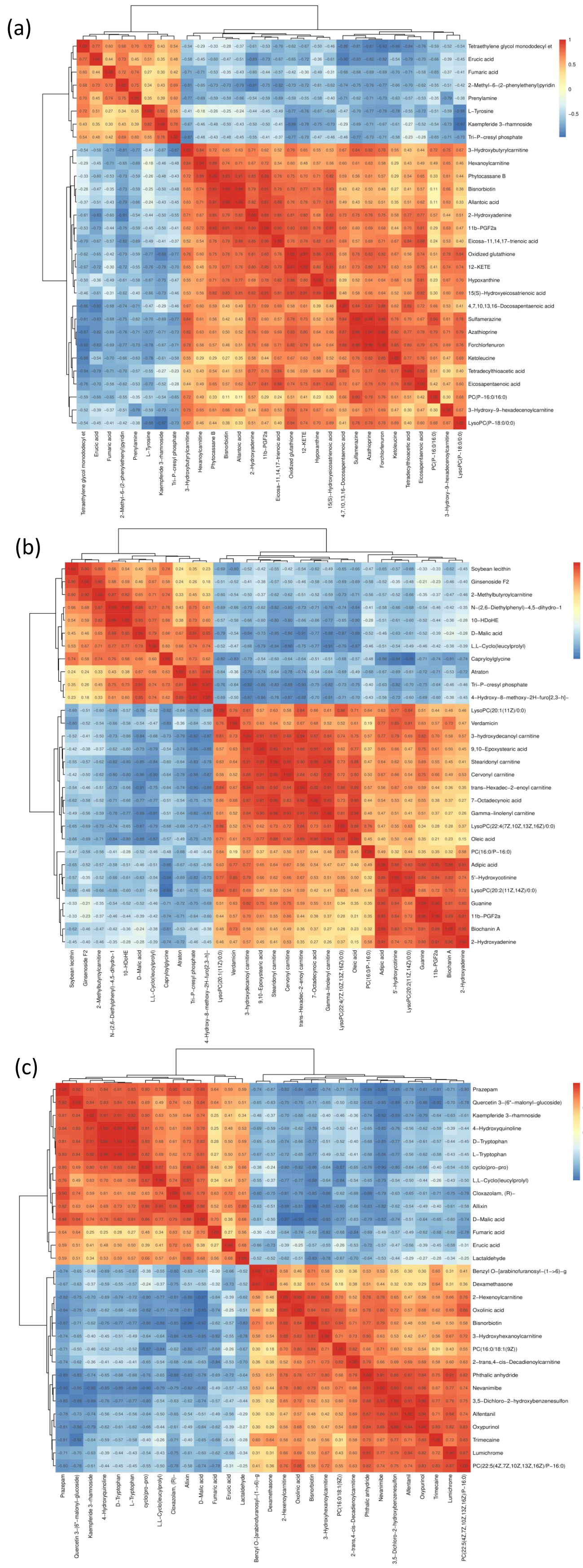

Fig S13. Muscle metabolite correlation analysis

a. Muscle metabolite correlation analysis(24h vs CON)
b. Muscle metabolite correlation analysis(24h vs 72h)
c. Muscle metabolite correlation analysis(72h vs CON)

Table S1. Formulas for Calculating Pharmacokinetic Parameters Beyond Observed Concentration-Time Curve Fitting

Pharmacokinetic Parameters	Calculation Formula
$t_{1/2}\alpha$	$t_{1/2}\alpha=\frac{ln2}{\alpha}$
$t_{1/2}\beta$	$t_{1/2}\alpha=\frac{ln2}{\beta}$
K10	$\frac{\alpha\beta}{K21}$
K12	$K_{12}=\alpha+\beta-K_{21}-K_{10}$
AUC(0-∞)	$AUC(0\text{-}\infty)=AUC(0-t)+\frac{Ct}{\lambda_z}$

Table S2. Method recovery rate of OXO in various tissues of *Micropterus salmoides.*

Organization	Brain	Liver	Intestine	Kidney	Muscle	Plasma
Recovery Rate %	≥88.33	≥69.70	≥77.10	≥81.32	≥88.55	≥80.23

Table S3. XCMS main parameters

Peak extraction is primarily achieved using the open-source software XCMS. The process includes steps such as peak alignment, peak picking, normalization, deconvolution, and compound identification. The main parameter settings for peak extraction and identification are shown in the table below.

Item	Parameter
method	centWave
minfrac	0.5
snthr	6
ppm	30
peakwidth	5,25
bw2	5
mzwid	0.015
mzdiff	0.01
profStep.OBIWarp	0.1

Table S4. MetaX main parameters

Post-processing of extracted peaks primarily includes primary metabolite identification and quantitative analysis.

1. Primary identification metaX parameters

Item	Parameter
adduct ion	pos: [M+H]+,[M+Na]+,[M+K]+,[M+NH4]+<br>neg: [M-H]-,[M+NH4-2H]-,[M+2Cl]2-,[2M-3H]3-
ms1 mass tolerance	10 ppm
database	Depending on the project, the specific databases used can be found in the analysis results.

2. Secondary identification parameters

Item	Parameter
ms1 mass tolerance	0.01 Da
ms2 mass tolerance	0.05 Da
identification score cut off	75%
database	in-house, Massbank, HMDB, Lipidblast

3. Metabolite quantification metaX parameters

Item	Parameter
missing value imputation	knn
scaling	pareto
normalization	pqn
